# Adaptive deep conditional random field with blockchain for secure data sharing in software-defined wireless body area networks

**DOI:** 10.1038/s41598-026-49079-w

**Published:** 2026-05-25

**Authors:** Sayila Subrahmanyam, Rajesh Arunachalam, Surendra Kumar Shukla, Amit Arora, Sumanth Venugopal, Thella Preethi Priyanka

**Affiliations:** 1 Department of Electronics and Communication Engineering, Saveetha School of Engineering, Saveetha Institute of Medical and Technical Sciences, Thandalam, 602105 Chennai, Tamil Nadu India; 2 School of Computer Science Engineering and Technology , Bennett University, Greater Noida, Uttar Pradesh 201310 India; 3 Department of Electrical, Electronics and Communication Engineering, Galgotias University, Greater Noida, Uttar Pradesh 203201 India; 4 Manipal Institute of Technology Bengaluru, Manipal Academy of Higher Education, Manipal, India; 5 Department of Computer Science and Engineering, Koneru Lakshmaiah Educational Foundation, Vaddeswaram, Guntur, Andhra Pradesh 522502 India

**Keywords:** Software Defined Wireless Body Area Network, Adaptive Deep Conditional Random Field, Enhanced Piranha Foraging Optimization Algorithm, Optimal Key-based Multi-Authority Attribute-Based Encryption, Computational biology and bioinformatics, Engineering, Mathematics and computing

## Abstract

Presently, blockchains are widely employed to execute the secure data transmission process among users. However, sharing the sensitive information about the patients among multiple users in the healthcare sector is difficult due to integrity and confidentiality issues. Thus, to tackle these issues in prior models, a secure data-sharing framework for Software-Defined Wireless Body Area Networks (SDWBANs) is designed. In order to ensure privacy and controlled access in SDWBANs, blockchain technology and encryption techniques are considered, which help to protect sensitive medical data. Then, the Adaptive Deep Conditional Random Field (ADCRF) is employed to perform the decision-making procedure. Further, an advanced encryption technique, Optimal Key-based Multi-Authority Attribute-Based Encryption (O-MA-ABE), is employed to ensure that authorized users can access the confidential health data. Here, the Modified Escape Search-based Piranha Foraging Optimization Algorithm (MES-PFOA) is employed to tune the Hyperparameters of ADCRF and keys of O-MA-ABE. Then, the overall performance of the developed framework is compared with classical approaches using metrics such as computational time, decryption time, and decision-making accuracy. In various validations, the developed MES-PFOA-O-MA-ABE + ADCRF-based data sharing model accomplished higher accuracy as 98.7%, precision as 98.3%, minimal encryption time as 210 (ms) and throughput as 275 (TPS) than the recent data sharing models like DTAC-TL-QM, SCCE-DS, BFL-hIoT and PPFL-ICP.

## Introduction

Wireless Body Area Networks (WBAN) consist of sensor devices that are deployed on the human body to gather physiological information related to body functions. These sensors continuously monitor the sensitive information of the patients and transmit the gathered information to server gateway nodes within the WBAN architecture^1^. In the healthcare sector, WBANs enable medical professionals to remotely monitor patients in home environments and assess their physical conditions^2^. In addition to clinical applications, WBAN allows medical professionals to assess the health condition of athletes, where the coaches analyse the variation in athletes’ physical conditions during training sessions to regulate training intensity and enhance performance^3^. The WBAN system comprises multiple sensors for measuring the physiological parameters, such as respiratory rate, Electrocardiogram (ECG) levels, glucose level, and blood pressure^4^. These sensors are usually placed on the body surface and communicate via the gateway device using short-range wireless communication technologies like WiFi, Bluetooth, and ZigBee^5^. The gateway devices subsequently transfer the gathered patient’s information to the cloud environment, where the healthcare professionals can access, analyze and utilize the stored information for the decision-making process^6^. In certain scenarios, WBANs are also employed to analyze healthcare information and suggest preventive measures to address the medical challenges^7^.

In many medical applications, the gathered physical data are more significant than others and require prioritized analysis to provide timely clinical decisions^8^. However, the open nature of WBAN environments and the limited trustworthiness of network servers make the sensitive data vulnerable to security threats and unauthorized access^9^. To solve these challenges, Software-Defined Networking (SDN) are designed, which is a promising paradigm that enables centralized control and flexible network programming by decoupling the control plane from the data plane^10^. Despite its advantages, SDN introduces several security challenges due to the lack of robust access control and authentication mechanisms between the centralized controller and network devices. The absence of effective security protocols at the interface layers enables malicious users to gain access to the SDN network, thus affecting the functions of the controllers and network operations^11^. Hence, there is a necessity to develop an efficient, secure data sharing mechanism to protect the application-controller in an SDN-enabled healthcare environment^12^.

Blockchain technology has attained significant attention in recent days for providing higher security, trust and transparency in distributed healthcare systems. Blockchain-based techniques reduce the need for centralized entities and mitigate the risk associated with malicious users while maintaining data availability and integrity^13^. Because of the decentralized nature of the blockchain, it ensures that all the participants maintain a copy of the ledger to improve resilience against attacks^14^. Blockchain follows the concept of the Peer-To-Peer (P2P) model, which helps to enhance the availability and reliability and highly minimized the chance of single-point failure^15^. The integration of blockchain in Software-defined WBAN (SWBAN) provides secure data sharing, where the data owner has full control over their sensitive data^16^. The developed SWBAN paradigm combines the principles of SDN into WBANS by separating the data and control planes, which enables centralized management, and it is effective in handling a huge volume of distributed health data. It is frequently involved with the lengthy validation process, and hence it fails to adequately guarantee the data authenticity and integrity^17^. With the growing number of patient sensors, the volume of healthcare data introduces additional complexity and latency while storing the data in a blockchain. The resources needed for performing secure data storage in the cloud are also a challenging process. To resolve these limitations, it is essential to design a secure and efficient data-sharing model with the combination of machine learning and blockchain technology in SDWBAN systems.

The contributions of the designed secure data-sharing model are listed here.

- To design an innovative data-sharing model for SDWBAN by integrating the function of blockchain, deep learning and encryption approaches. The prime intent of the developed model is to address the privacy and security limitations of existing SDWBAN systems by neglecting the necessity of the mediator and enabling the patients to have complete access to their healthcare records.
- By combining the blockchain and SDWBAN models provide robust healthcare data protection against data breaches, unauthorized access and tampering. Blockchain’s decentralized ledger protects SDWBANs from single-point failures, data tampering, and unauthorized modifications. The integration of encryption in blockchain ensures secure and verifiable data exchanges.
- To construct an effective decision-making model named ADCRF that permits only the authenticated user to access the data. Moreover, the decision-making procedure is enhanced by optimizing the system parameters of the Deep Conditional Random Field (DCRF) system, like the number of hidden neurons, epochs and learning rate, by the implemented MES-PFOA that helps to improve CSI and to reduce FNR. This decision-making process reduces the risk of unauthorized access to sensitive information in SDWBANS.
- To develop a novel encryption technique named O-MA-ABE to preserve the healthcare records of the patients from unauthorized access. In O-MA-ABE, an optimal key is selected by the designed MES-PFOA to reduce the time and memory size of the encryption process. This model can effectively manage access to the user information according to the healthcare attributes and also guarantee that only authenticated users have the right to access or utilize the sensitive data stored on the blockchain.
- To introduce an adaptive tuning approach termed MSE-PFOA to perform parameter optimization during decision making and optimal key selection during the encryption process. In the decision-making process, MSE-PFOA is employed to optimize the hyperparameters within the DCRF. Moreover, during the encryption phase, this model is utilized to choose an optimal key for performing encryption procedures. The fitness-based concept is introduced in the proposed MSE-PFOA to provide better solutions with a faster convergence rate.

The layout of this research is given here. The limitations and features of the standard data-sharing systems are explained in 2nd part. The overview of the developed framework, along with the data initialization is provided in 3rd part. The development of access control and decision models is given in the 4th part. The description of the encryption technique, along with the tuning approach, is presented in the 5th section. Finally, the result and conclusion of the suggested system are provided in the 6th and 7th parts.

## Literature survey

### Related works

In 2022, Hasan et al*.*^18^ presented an efficient model by integrating blockchain with SDWBAN for ensuring secure data transmission. Additionally, they introduced and combined smart contract-aided fine-grained access control rules to guarantee that only the information owner has the right to manage the medical information. The simulation results revealed that the suggested framework has attained better throughput as well as experiences minimum overhead with respect to latency than classical cloud-aided techniques.

In 2022, Rani et al*.*^19^ introduced a novel architecture for smart cities through integrating the benefits of SDN along with blockchain. Moreover, this model was categorized into diverse fields, where SDN was employed to determine the possible threats and transfer the preserved information towards the blockchain. Additionally, experimental evaluation based on performance measures revealed that the suggested system has attained greater competence than standard approaches.

In 2021, Abbas et al*.*^20^ designed a lightweight blockchain-aided authentication protocol that preserved the information of the sensor. In addition, the path evaluation was executed by employing a genetic strategy-based SDN controller that was employed for on-demand routing for tuning the node’s energy utilization within the Internet of Things (IoT). Additionally, a Route Correctness Mechanism (RCM) was generated to verify the occurrence of malicious nodes along the estimated path. The suggested network was estimated with traditional approaches to showcase its efficiency.

In 2022, Algarni et al*.*^21^ introduced a unique comprehensive privacy model to preserve the application–controller interface. In addition, they suggested a controller-independent lightweight blockchain framework and explored the blockchain’s privacy characteristics by minimizing the computational overhead of the blockchain. Here, the controller validated the device automatically and the details of the SDN controller by utilizing token-aided authentication. The simulation outcomes revealed that the developed controller can effectively safeguard the NBI, according to the basic security goal, while creating irrelevant overhead.

In 2021, Derhab et al*.*^22^ suggested Blockchain-based Multi-Controller with SDN (BMC-SDN), a security framework that combined blockchain as well as multi-controller SDN and categorized the system into multiple fields. Every SDN domain was handled by a single master controller, which interacted via blockchain. Furthermore, the master controller generated blocks of system flow upgraded, and the unnecessary controllers evaluated the latest block according to the suggested reputation protocol. The simulation outcomes proved a rapid and best detection of fake flow rule injection.

In 2022, Zeng et al*.*^23^ introduced a blockchain-aided system for guaranteeing safe routing between several domains within SDN-aided IoT models labeled as Blockchain-based SDN-aided Secure Routing (BSDNSR). Additionally, they employed the theory of reputation, which comprised both local and global reputation for preserving the reliability of routing, and the global reputation was kept within the blockchain. The evaluation outcomes proved that the suggested approach successfully constructed trust among several controllers as well as guaranteed safe routing among different fields.

In 2023, Sanjith et al*.*^24^ have suggested a unique approach, which integrated blockchain and effective encryption approaches along with WBAN. In addition, a modified strategy was proposed for signature encryption within WBAN applications. Additionally, a Hyperelliptic curve-aided approach was employed with an authenticated certificate-less signature key encryption technique that assisted in addressing numerous threats, including Man-In-The-Middle (MITM), fundamental impersonation threats, and key offset threats. The suggested approach was analyzed with conventional techniques to monitor its competence.

In 2021, Sebbar et al*.*^25^ developed a defense protocol that determined and minimized the effects of threats according to effective machine learning approaches. Data pre-processing and learning phases were executed by employing the constructed database offered to the SDN. The resultant values revealed that this system has the capacity to resolve sniffing threats, which can be identified and prevented rapidly. At last, they attained better accuracy along with a minimum false-positive detection of attacks.

#### Recent techniques for secure data sharing

In 2024, Kumar and Ali et al*.*^26^ implemented an authentication protocol in 6G with Internet of Nano Medical Things (6G-IoNMT) communication systems using a smart contract paradigm. The real-time information has been effectively managed with minimal latency, which has been accomplished through decentralized P2P cloud servers. The performance of this authentication protocol has been validated through thorough security and privacy assessments, which included Scyther tool analysis, model evaluations, and informal security evaluations. The experimental results ensured that the effective authentication performance of the developed approach in terms of measures like energy consumption, security and efficiency.

In 2025, Prajapat et al*.*^27^ presented a quantum-safe blockchain technology with the utilization of lattice-based encryption. This technique used the consensus nodes for performing data encryption, and it effectively removed the reliance on the consensus process. The performance assessment in terms of security measures ensured the effective performance against probabilistic polynomial-time adversaries. The communication cost of the model has been greatly minimized and provided better robustness in an IoT environment.

In 2024, Ali et al*.*^28^ presented a decentralized authentication framework to safeguard sensitive data from unauthorized access, which provided secure cloud-based data storage. The developed decentralized model prioritized transparency, robustness, and energy efficiency. Experimental results ensured the solution’s resilience across diverse malicious attacks.

In 2024, Naik et al*.*^29^ have designed an efficient mechanism by incorporating ensemble deep learning models to verify the latent patterns. In order to improve the trust score, the Dynamic Trust-Aware Controller (DTAC) was used, which validated the historical behaviours. Further, decision-making was performed through Threshold-based Logic with a Quarantine Mechanism (TL-QM). In the validation, higher scalability was accomplished over classical models.

In 2024, Chen et al*.*^30^ have proposed a Secure and Controllable Cloud–Edge Collaborative Data Sharing (SCCE-DS) mechanism for ensuring access control. SCCE-DS was efficient in accelerating the user requests and eliminating the validation complexity. In the security analysis, the developed SCCE-DS accomplished better performance by eliminating the delay.

In 2024, Ganapathy et al*.*^31^ have suggested Blockchain-enabled Federated Learning for healthcare IoT (BFL-hIoT), which supported for secure communication. Moreover, the BFL-hIoT verified various attacks in minimal time and accomplished higher accuracy in classifying the delay.

In 2024, Munusamy and Jothi^32^ have implemented Privacy-Preserving Federated Learning with Intelligent Control Policy (PPFL-ICP) for ensuring the privacy along with data security. PPFL-ICP was capable of handling the centralized attacks and preserving the overall privacy while transferring sensitive details. In the validation, higher security and robustness were maintained over different classes.

#### Critical synthesis on literature works with its research gaps

In healthcare systems, the researchers focus mainly on the integration of blockchain and SDN-based structures for providing higher trust, security and access control. The studies mentioned in the literature works demonstrate that the integration of blockchain with SDN models enhances the ability of authentication, data transmission and routing by exploring decentralized control and smart contract mechanisms. Lightweight authentication mechanisms have been developed to decrease the communication and computation overhead in healthcare environments. These techniques mainly depend on static security policies, which limit their adaptability in dynamic network conditions. The security of the healthcare environment is strengthened by the adoption of advanced cryptographic techniques, such as lattice-based encryption and hyperelliptic curve cryptography techniques. These cryptographic techniques do not meet the performance trade-offs, including controller-side overhead, throughput degradation and blockchain validation delays. The scalability of the traditional blockchain-based paradigms is limited by simultaneous access requests. Current cryptographic approaches are highly vulnerable to quantum attacks, which affect the data integrity. Hence, an integrated approach is needed to provide better balancing of the network adaptability, efficiency and security.

The features and the limitations of data transmission models in SDWAN are given in Table 1.

**Table 1:** Features and challenges of standard data transmission techniques in SDWAN.

Author [citation]	Methodology	Features	Challenges	Solution By Developed Framework
Hasan et al*.*^18^	Automated Validation of the Internet Security Protocols and Applications (AVISPA)	This model is more effective in addressing the problems due to the confidentiality of the data, as well as the integrity This model guarantees that only the authorized user can transmit private information in the absence of data owners	This technique presents less efficiency when applied in the public blockchain	In order to improve the efficiency of blockchain, ADCRF is employed to verify all the transactions in all the protocols and provides better decisions. Further, decentralized trust in the network is improved using O-MA-ABE
Rani et al*.*^19^	Distributed Ledger Technology (DLT)	This model can determine and prevent attacks within the blockchain This technique has improved bandwidth and minimized energy utilization	This model lacks the capacity to resolve the risk of a single point of failure	These issues are eliminated using O-MA-ABE, which distributes equal power among different independent nodes. In case, one authority node fails remaining nodes continue the access managing
Abbas et al*.*^20^	RCM	This is more effective in determining the best path on the SDN for information transmission and guarantees better security for the transmitted information	The robustness of the model is influenced by the absence of meta-heuristic approaches	These issues are tackled by tuning the keys of O-MA-ABE, which helps to select the best routes to deliver the data packets to the user
Algarni et al*.*^21^	BCNBI	This model improves the security of the system by minimizing the overhead and delay	This model lacks the capacity to resolve the problems associated with data consistency and security	In order to rectify the security-related issues, O-MA-ABE is selected, which ensures authorized access. Moreover, ADCRF is efficient in detecting malicious activities
Derhab et al*.*^22^	BMC-SDN	It can determine the injected flow by maintaining a minimum detection delay This model allows the malicious controller’s detection time based on the needs of the system manager	This model is not capable of resolving the security issues within the southbound interfaces	The developed ADCRF is efficient in identifying false data inclusion, and also the security-related issues are eliminated by considering the authenticated authorities using O-MA-ABE
Zeng et al*.*^23^	BSDNSR	This technique guarantees safe and secure routing among different domains The reliability of the routing is improved by employing the global and local reputation protocols	This technique struggles to address scalability issues, especially for huge-scale IoT devices	In order to eliminate the scalability issues, ADCRF is used, which offers better decisions for fulfill the user requests by analyzing the learned patterns. Moreover, routing overhead issues are tackled through O-MA-ABE
Sanjith et al*.*^24^	Hyperelliptic curve-based technique	This model is more effective in determining the risk of unauthorized access and various system attacks This model is more effective in addressing the issues related to certificate management and key escrow	This method is sensitive towards unauthorized access due to the absence of multi-factor authentication	In order to tackle these issues, multiple independent authorities are employed with the help of O-MA-ABE. Further, identity-aided verification is performed through ADCRF to maintain the access requests
Sebbar et al*.*^25^	Random forest (RF)	This system can analyze and prevent the anomalies that influence the transmission procedures within the SDN	This model demands a hybrid model to boost the network efficiency	In order to enhance the overall network efficiency, an advanced ADCRF is included. ADCRF is efficient in tackling the overall complexity and improving validation speed

### Problem statement and solutions

The key limitations of the SDWBAN models are the sensitive nature of the data and the limited computational capability of WBAN devices.

- The classical SDWBAN approaches lack intelligent decision-making mechanisms regarding access control over sensitive data, resulting in decreased network efficiency and increased system latency in detecting security threats. Latency issues in the network are eliminated using O-MA-ABE with the help of optimal keys. Moreover, it speeds up the overall processing through quick operations.
- The traditional cryptography-based approaches are mainly employed to enhance the healthcare data integrity, where most of the approaches mainly depend on single authority key management approaches, which are highly sensitive to insider threats. Hence, to eliminate the security-related issues and attacks in the developed secure data sharing model, ADCRF is used, which verifies that the transmitted messages do not include any secret information and also eliminates data breaches.
- Most of the research works do not focus on the access control mechanisms, which affect the reliability and robustness. Higher computation cost and overhead are significant issues in conventional secure data storage mechanisms. Thus, to tackle the computational overhead issues, an advanced encryption technique, O-MA-ABE is used, which helps to eliminate the latency issues.
- The energy consumption and communication overhead of the traditional approaches are higher, which affects the scalability of the model in practical healthcare cloud applications. Here, the O-MA-ABE is employed to tackle the energy consumption issues at the data transmission phase, along with better communication being maintained under dynamic conditions. Further, the scalability-related issues are tackled through the developed ADCRF, which helps to maintain higher robustness and eliminate the latency issues.

*Novelty.* This work presents an adaptive privacy-preserving module for providing higher security to healthcare data. This work introduces an ADCRF model for enabling context-aware decisions regarding access control. The developed ADCRF module dynamically captures the temporal and spatial dependencies from the WBAN data, which significantly enhances the threat detection capabilities. The attribute-based encryption standard is developed to address the vulnerabilities of traditional encryption schemes, which helps to ensure that only authorized entities can decrypt the sensitive clinical information. The utilization of multiple authorities in the developed model eliminates the issues with single points of failure and highly preserves the sensitive information from malicious users. Furthermore, the optimization of ADCRF hyperparameters using MES-PFOA boosts the decision-making ability with highly minimized computational overhead, which enables the network to be highly suitable for resource-constrained SDWBAN systems.

## Novel blockchain-enabled secure data sharing in software-defined wireless body area networks

### General view on software-defined wireless body area network

SDWBAN includes the function of SDN. Similar to the classical WBAN model, the SDWBAN comprises three planes, namely, the application plane, control plane and data plane.

*Application plane.* This plane is placed on the uppermost level of the network, and it is linked with the management authority. This plane assists the medical provider in analyzing the condition of the patients. Moreover, they can be employed to define, install, execute and handle the latest WBAN device.

*Control plane.* This plane can manage the entire system’s functions and learn the overall architecture of the system. This plane transmits data packets for delivering the commands towards the SDN-aided switches (SDESW). In addition, the SDESW upgrades their flow table according to the commands. Furthermore, the interaction channel within the application and control plane is defined as the Northbound Interface (NBI), in which the Southbound Interface (SBI) functions as an intermediate control and data plane.

*Data plane*: This plane comprises switches and sensors. The sensors within the system are responsible for transmitting the information to the SDESW, and every SDESW is linked with the local controller. Furthermore, according to the queries, the switches transmit the data packets to the respective receiver. Here, the Body Sensors (BS) connected to the patients create a cluster, where every participant within the cluster shares a particular SDWBAN for processing.

### Data sharing security problems in SDWBAN

The data-sharing model within the SDWBAN struggles due to certain challenges that affect the performance of the model during safe and robust data transmission, and they are discussed here.

*Patient-centred framework.* In clinical cases, it is significant to create an innovative and effective data transmission medium. Due to the absence of coordination among the collaborators, the efficacy of the model during data transmission is influenced. Moreover, both the patients and the providers face various issues within the data collection and transmission procedures. Primarily, authorization from the patients is necessary to suggest the appropriate treatment plan. Thus, the patient-driven model creates various limitations related to data privacy, governance and incentives.

*Data Management, Security and Anonymity.* In SDWBAN, managing the data transmission throughout the system is a significant task. Trust is the most important factor during data handling, in which patients have to safeguard their private data from unauthorized access and ensure their privacy.

*Integrity of the information and Storage issues.* The SDWBAN models hold limited storage spaces. It is challenging to preserve all information related to the patients, which comprises medical records, images, and lab reports, which demands a huge volume of storage space. In these cases, the private information is preserved on the public cloud storage, which affects the truthfulness of the preserved information.

### Developed blockchain-enabled secured data sharing model in SDWBAN

A blockchain-enabled data transmission model is designed to address the limitations faced by the SDWBAN model by neglecting the necessity of the mediator and permitting patients to have complete access to their healthcare reports. Moreover, they are more effective in overcoming the challenges associated with data security, integrity and management. During the implementation phase, the system attributes, namely WBAN attributes, Blockchain attributes and transaction attributes, are initialized at the beginning. Further, access control is made by employing the blockchain, which is significant to prevent the entry of unwanted access to the data. Then, the decision-making procedure is implemented via the designed ADCRF model. Furthermore, the decision-making performance is increased by tuning the hyperparameters within the DCRF using the implemented MES-PFOA. Here, the hyperparameters like the hidden neuron counts, epochs and learning rate are optimized to increase the CSI and reduce FNR. Additionally, the privacy of healthcare data is guaranteed by performing data encryption. Here, the designed O-MA-ABE approach is utilized to encrypt the data. Moreover, at the encryption phase, an optimal key is selected by the implemented MES-PFOA technique that assists in minimizing the time and memory size. Furthermore, the O-MA-ABE technique is employed to decrypt the information for future use. At last, a set of investigations is implemented to evaluate the competence of the designed data-sharing framework over traditional models.

The developed model combines the blockchain, deep learning architecture and advanced encryption method, which is not explored in the traditional SDWBAN models. The adoption of the blockchain paradigm ensures decentralised data management for enhancing trust in healthcare applications. The ADCRF model allows context-aware decision-making related to network behaviour prediction and privacy management. This model learns the network behaviour from the collected data, and it is adaptable to changing network topologies. The optimized encryption scheme is helpful to ensure that only authorized entities can decrypt the sensitive medical data. The combination of optimal key management with a deep learning-based access control mechanism reduces the computational overhead and ensures energy efficiency.

The step-by-step process of the proposed model has been provided in the following points.

- **Data Collection from WBAN Sensors:** Wearable sensors continuously monitor the healthcare attributes of the patients, such as heart rate, blood pressure and glucose levels.
- **Transmission to SDN Controller:** Sensor data is transmitted to the SDN controller via secure wireless links. The SDN controller performs central decision-making for better data communication.
- **ADCRF-based Decision Making:** The controller applies the collected data to the ADCRF model. The developed ADCRF model analyzes the data for anomalies, inconsistencies, or abnormal patterns to ensure trustworthiness. System parameters of ADCRF are optimized via MES-PFOA to maximize accuracy and minimize false negatives.
- **Encryption via O-MA-ABE:** Verified data is encrypted using O-MA-ABE, which provides fine-grained access control. Multiple authorities distribute attribute-based keys, thus guaranteeing scalable and secure access for legitimate users only.
- **Blockchain Integration for Secure Storage:** The encrypted data, along with its cryptographic hash, is sent to the blockchain ledger. Blockchain ensures immutability, traceability, and tamper-proof storage of the health data. Authorized users send data access requests via smart contracts on the blockchain.
- **Data Retrieval and Decryption:** If access is authorized, the encrypted data is provided to the requester. The user decrypts the data using attribute-based keys, which helps to enable only legitimate entities to access the sensitive health records. The developed system continuously monitors the SDWBAN for new anomalies or security threats.

**Fig. 1:**
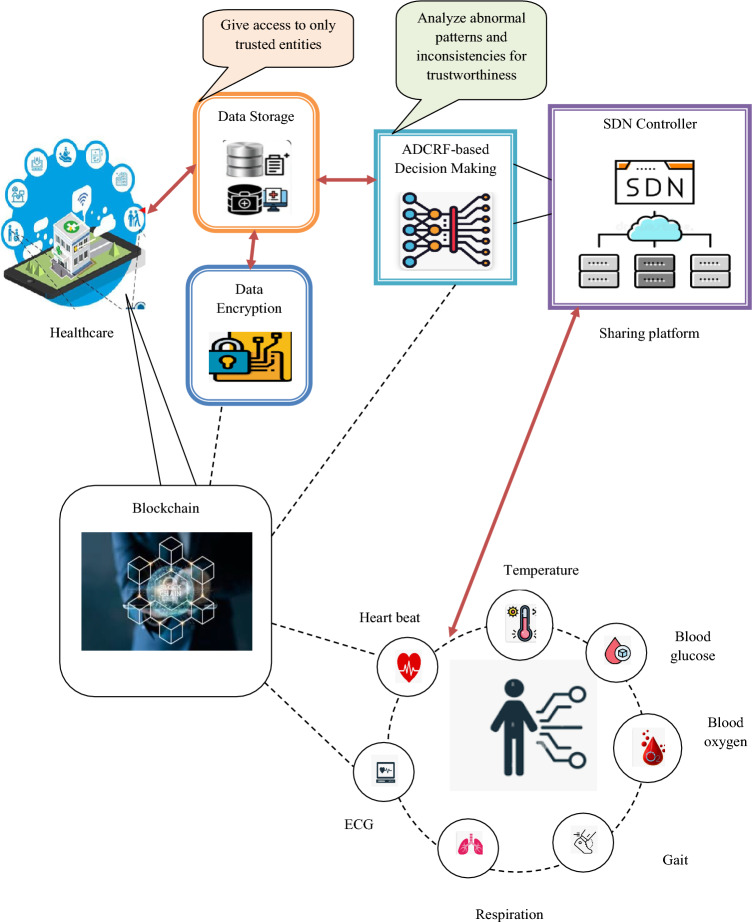
Pictorial Illustration of a Designed Blockchain-enabled Secured Data Sharing Model in SDWBAN.

The pictorial representation of the designed blockchain-enabled secured data-sharing system in SDWBAN is given in Fig. 1.

### Dataset description and SDWBAN attribute initialization

The SDWBAN attributes employed for the execution of the designed blockchain-enabled secured data-sharing model are listed below.

- WBAN attributes include range of communication, frequency of data transmission, data rate, topology of the system, power consumption, sensor type, latency, and bandwidth.
- Blockchain attributes include the block header, merkle root, timestamp, the hash of the prior block header and transactions.
- Transaction attributes include the private key of the user, the hash, the public key of the next user, the signature of the user, and the contents.

The dataset needed for performing secure healthcare data transfer is attained from the link: “https://www.kaggle.com/datasets/akshaydattatraykhare/diabetes-dataset” with access date 23–01-2026. The name of the dataset is Diabetes Dataset, which is taken from the National Institute of Diabetes and Kidney Diseases. This is a publicly accessible dataset. This dataset is generally used to predict whether the patient has diabetes. This dataset includes attributes such as insulin level, blood pressure, glucose level, age, skin thickness and BMI. From this dataset, 25% of the dataset is used for testing, and 75% of the dataset is used for the training process.

In this research work, the diabetic dataset is employed to display the physiological data samples of the patients generated through the wearable medical sensors. Then, these data samples are transmitted to the SDWBAN or WBAN-based healthcare monitoring systems. Furthermore, sensitive diabetic medical samples are analyzed for secure access control, transmission and privacy protection. Moreover, the medical attributes with the sensor-generated patient data are transmitted securely and accessed in the network. Hence, the diabetic dataset is suitable to verify the decision-making and encryption efficiency in the developed security-aware technique under real-world monitoring conditions. Moreover, these sensitive data samples of the individuals are securely stored in the blockchain.

## Blockchain technology for access control in SDWBAN and adaptive deep conditional random field for decision making

### Data generation for blockchain access

The blockchain type used in the model is a permissioned blockchain with the platform modelled as Hyperledger Fabric–like architecture. The consensus mechanism used is Practical Byzantine Fault Tolerance (PBFT) with a Block Creation Interval of 2 s. The size of the block considered is 1 MB, the average transactions per Block is 200, and the Cryptographic Scheme used is O-MA-ABE. Moreover, smart contract support is also provided with access control logic.

The SDWBAN model is deployed with WBAN sensor nodes of wearable biomedical sensors (low-power), several sensors as 10–25 per WBAN, a communication protocol as IEEE 802.15.6, SDN Controller as Centralized controller (Edge Server), Deep CRF Execution is performed in SDN Controller (Edge Layer), Blockchain Nodes as SDN Controller + Healthcare Gateway Nodes, and data flow is in between Sensors → SDN → Blockchain → Cloud.

The WBAN often creates information for processing, and the volume of the information increases over time. Currently, blockchains are employed to preserve a huge amount of information. It is significant to create a smart data management protocol. Initially, the bio-medical information is accumulated from various sensors required to be transferred to the storage applications during a specific time period that is mentioned by the stacks of the controller. Moreover, the controller forwards the new information transmission suggestions to the SDWBAN. Later, the storage applications preserve the data patterns that comprise the patient’s name, ID, details of the doctor, position, and time. Then, offers a transaction towards the blockchain for upgrading the physical information of the patients. Furthermore, the patient’s physical information and the hash value of the preserved information are given to the blockchain to guarantee the reliability of the information.

*Process involved in data generation: *In the proposed SDWBAN framework, the WBAN sensors are deployed directly on the patient’s body to continuously acquire physiological data, which provides minimal computation requirements. The collected data is then forwarded to the WBAN gateway located at the patient side, which handles preliminary aggregation with low latency. The SDN controller, deployed on an edge server, functioned as the central control point, and it serves as the decision-making logic. The ADCRF model is applied on the SDN controller, which enables adaptive, near real-time inference for anomaly detection and access control. System parameter tuning is handled by the EPFOA optimizer, which operates offline or periodically at the SDN controller to optimize ADCRF parameters without adding runtime latency. For secure and auditable data storage, the blockchain ledger is distributed among controllers and gateways, which gives tamper-proof access control. Blockchain validators are implemented on SDN controllers to ensure energy-efficient and trusted consensus. Finally, O-MA-ABE encryption is executed at the SDN controller, providing secure, fine-grained access control for health data with acceptable cryptographic overhead.

### Blockchain technology for access control in SDWBAN

In the medical sector, the healthcare provider only has the right to utilize the physical information of the patients, which is allocated to them. The patients permit their allocated healthcare providers to access their medical records by creating a secure blockchain-aided access control list, which is executed by smart contracts. It is established in the blockchain by the patient to establish their rights to access the healthcare providers. In this framework, an access control matrix is employed for the execution of the access permission via smart contracts. Here, the access control matrix links the resources to the corresponding rights of access. Later, it describes who has the right to perform a particular task with the appropriate resources. Additionally, an Access Control List (ACL) protocol is introduced to the access control matrix framework. ACL technique is referred to as an object-centred method that describes access control rights for every object *P* using a list *A*. This list comprises the rights of access given to the data users. In order to assign ACL to every object within the network, they described a list that establishes a list of information on users and rights assigned to every object. In addition, this list is preserved within the blockchain on the smart contract that is utilized as a Data-Sharing Contract (DS-Contract).

Furthermore, the DD-Contracts are established and employed in the blockchain by every patient to describe the rights assigned to the data users. Thus, the patient is considered as the contract owner. In the ACL technique, every object is connected with a list, which describes the information of the user, and then access is given to them. Moreover, the DS-Contract demands a list of information users to be handled, and the access of the trusted users has been assigned to them. Moreover, in this designed model, access control is performed by employing blockchain technology. The prime function of this framework is to allow only the authenticated user or healthcare providers to access the healthcare information preserved within the blockchain, and unauthorized users are denied access by the model.

**Fig. 2:**
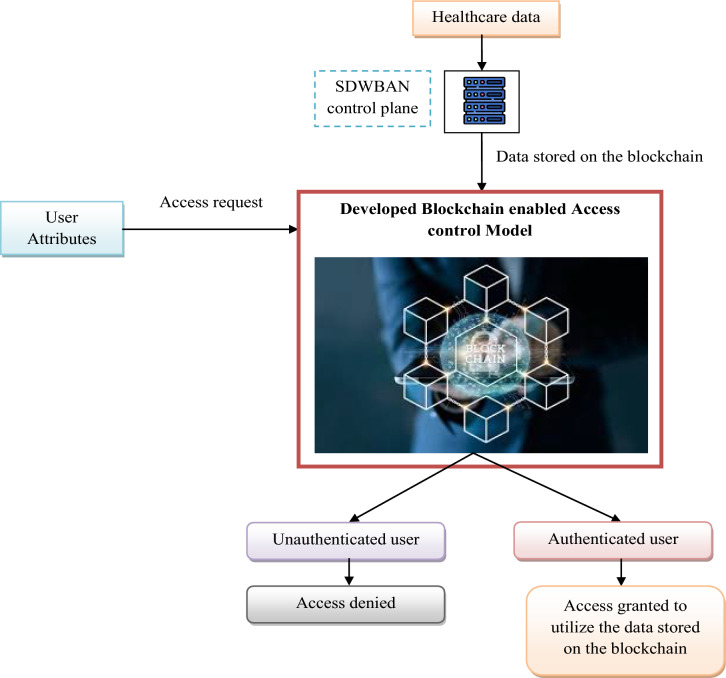
Developed Blockchain-aided Access Control Model.

The pictorial illustration of the developed blockchain-aided access control model is offered in Fig. 2.

### Developed ADCRF decision-making network

*Purpose of ADCRF*: In this proposed data-sharing model, the decision-making procedure for access control is performed by employing the designed ADCRF model.

*Design of ADCRF:* The developed ADCRF is an adaptive version of DCRF. The term adaptive refers to the adjustment of model parameters using the developed MES-PFOA, which supports varying system conditions and data distributions. These updated parameters directly influence the learning potential and generalization effectiveness of the DCRF model rather than performing modifications to the internal inference formulation.

DCRF^33^ integrates Conditional Random Field (CRF) along with the concept of parameter optimization to improve decision-making efficiency. The integration of the DCRF model is effective at capturing both non-linear and contextual representations, which is essential for making dynamic decisions regarding access control. The DCRF is constructed by adopting *E* layers of neural architecture that are expressed in Eq. (1).

1 $$\begin{gathered} E\left( {Q,\theta ,\omega } \right) = - \sum\limits_{c = 1}^{J} {\log \,f\left( {n_{c,1} , \ldots ,n_{{c,H_{c} }} |k_{c,1} , \ldots ,k_{{c,H_{c} }} } \right)} \hfill \\ \,\,\,\,\,\,\,\,\,\,\,\,\,\,\,\,\,\,\,\,\,\,\,\,\,\,\,\,\,\,\,\,\,\,\,\,\,\,\,\,\,\,\,\,\,\,\,\,\, + \lambda 2||\theta ||^{2} + \lambda 3||\omega || \hfill \\ \end{gathered}$$

In Eq. (1), the lower and upper-layer parameters are indicated as *ω* and *θ*, respectively. The term $n_{c,1} ,$ is the initial state factor and $n_{{c,H_{c} }}$ is the final state factor. The term *J* denotes the number of training samples, *Q* is the set of *J* training samples. Here, the energy function is specified as *E*, hyperparameters are represented as *λ 2* and *λ 3*, and different conditions are given as $k_{c}$.

CRF’s latent characteristics depend mainly on the factors *θ* and latent non-linear factors $k_{c} = \left\{ {k_{c,1} , \ldots ,k_{{c,H_{c} }} } \right\}$. Latent characteristics are achieved at the coding region, and they are defined using Eq. (2).

2 $$\begin{gathered} \log \,f\left( {n_{c,1} , \ldots ,n_{{c,H_{c} }} |k_{c,1} , \ldots ,k_{{c,H_{c} }} } \right) \hfill \\ = \sum\limits_{g = 2}^{{H_{c} }} {n_{c,h - 1}^{H} Xn_{c,h} } + \sum\limits_{h = 1}^{H} {\left( {k_{c,h}^{H} Rn_{c,h} + g^{H} n_{c,h} } \right)} \hfill \\ + n_{c,1}^{H} \pi + n_{{c,H_{c} }}^{H} \tau - \log \left( {M\left( {n_{c} } \right)} \right) \hfill \\ \end{gathered}$$

The term $k_{c}$ is the output with *E - 1* layers, a single sequence is given as $n_{c}$, transition dependencies among the sequence at the consecutive states are represented as $\sum\limits_{g = 2}^{{H_{c} }} {n_{c,h - 1}^{H} Xn_{c,h} }$, and weights are indicated as *X*. Observed inputs of *k* for the time *h* matched with the states *n* are offered as $k_{c,h}^{H} Rn_{c,h}$. Next, the linear biases at the stages are signified as $g^{H} n_{c,h}$. Further, the boundary condition of the initial stage is given as $n_{c,1}^{H} \pi$, the final stage is provided as $n_{{c,H_{c} }}^{H} \tau$ and the normalization term is given as $- \log \left( {M\left( {n_{c} } \right)} \right)$ in the above equation.

The neural architecture’s *E - 1* layer achieves the results with the non-linear mapping $k_{c}$. Moreover, the composite function of feature vectors of DCRL is expressed in Eq. (3).

3 $$k_{c} = l_{E - 1} \circ l_{E - 2} \circ \cdots \circ l_{1} \left( {s_{c} } \right)$$

In Eq. (3), the composition operation is defined as *∘*, the logistic function is indicated as $l_{c}$ and parameter regulation is executed by employing $\theta \,\,and\,\,\omega$, in which $\omega = \left\{ {R_{e} |e \in \left[ {1, \ldots ,E - 1} \right]} \right\}\,\,\,and\,\,\,\theta = \left\{ {X,R,\pi ,\tau ,g,r} \right\}$. Initial inputs are specified as $s_{c}$, layers are given as $l_{1}$, and feature vectors are represented as $k_{c}$.

The non-linear representations from the model are effectively captured using this DCRF model.

*Working of ADCRF:* During the execution phase, the initialized system attributes such as access privileges, user credentials and contextual information related to access control are applied to the developed ADCRF model. The DCRF model is capable of learning rich contextual representations from the attributes, which leads to enhancing the decision-making ability. Moreover, the decision-making procedure is enhanced by optimizing the system parameters like hidden neuron count, epochs and learning rate of DCRF by the implemented MES-PFOA that helps to improve CSI and reduce FNR. The hidden neuron parameter optimization process controls the learning ability of the DCRF models, the number of epochs calculates the extent of training numbers and the optimization of the learning rate is helpful to update the weights of the model. Thus, helps to reduce the computational complexity, and the improper selection of these attributes leads to overfitting, which greatly affects the decision making ability. Continuously selecting hyperparameters for the optimization process is helpful in varying access control environments. Therefore, the authorized user has the only access to visualize the data, and hence the security of the model is protected from unauthorized access.

*Objectives of ADCRF: *In the developed ADCRF-based decision-making model, various parameters of DCRF, like hidden neurons, epoch and learning rate, are tuned using MES-PFOA in specific ranges. Here, parameter tuning supports enhancing the CSI and eliminating the FNR to offer better outcomes at the decision-making phase. The objective function of the developed ADCRF-based decision-making model during the decision-making procedure is given in Eq. (4).

4 $$Obj1 = \mathop {\arg \min }\limits_{{\left\{ {Hn_{f}^{DCRF} ,Ec_{g}^{DCRF} ,Lr_{h}^{DCRF} } \right\}}} \left( {\frac{1}{CSI} + FNR} \right)$$

Here, the number of hidden neurons that are selected within the limit $\left[ {5,255} \right]$ is indicated as $Hn_{f}^{DCRF}$, the number of epochs selected within the range $\left[ {5,50} \right]$ is denoted as $Ec_{g}^{DCRF}$ and the learning rate, which is taken from the range $\left[ {0.01,0.05} \right]$ is indicated as $Lr_{h}^{DCRF}$. In the ADCRF, other parameters like batch size are selected as 32, steps per epoch are chosen as 30, and the Adam optimizer is used for obtaining better decisions. Here, the term *CSI* specifies the CSI, which needs to improve and *FNR* represents the FNR, which needs to be reduced.

*Need for Optimizing hyperparameters:* In the developed ADCRF, an adaptive concept is considered by dynamically adjusting the hyperparameters in DCRF. Here, the hidden neuron counts in DCRF are tuned using MES-PFOA, which helps to maintain better balancing while learning the non-linear and complicated features, which helps to eliminate the overfitting and underfitting issues. In addition, pixel-level features are refined to optimize the feature representation. Moreover, the unbalanced hidden neurons in the network lead to instability issues at the training phase, so a proper tuning procedure is essential to stabilize the network learning efficiency. In order to maintain higher generalization, epochs in DCRF are tuned through MES-PFOA, which supports to ensure the overall efficiency of the validation dataset. Selecting the epoch in a particular range during the training phase helps to optimize the training time and also tracks the training and testing performances. Further, the learning rates are optimized to handle the non-convex details in the search space. Achieving a minimal learning rate helps to ensure the complicated joint probability at the label sequence and affects the convergence speed. In order to eliminate the fluctuation and losses, the learning rate is optimized through MES-PFOA, which helps to ensure stability at the training phase.

- CSI is the measure employed to estimate the accuracy of the decision taken, and it is numerically defined in Eq. (5).
  5 $$CSI = \frac{{R_{p} }}{{\left( {R_{p} + G_{n} + G_{p} } \right)}}$$
- FNR is the ratio of accurate decisions, which are wrongly classified as false by the system, and it is derived using Eq. (6).
  6 $$FNR = \frac{{G_{n} }}{{R_{p} + G_{n} }}$$

**Fig. 3:**
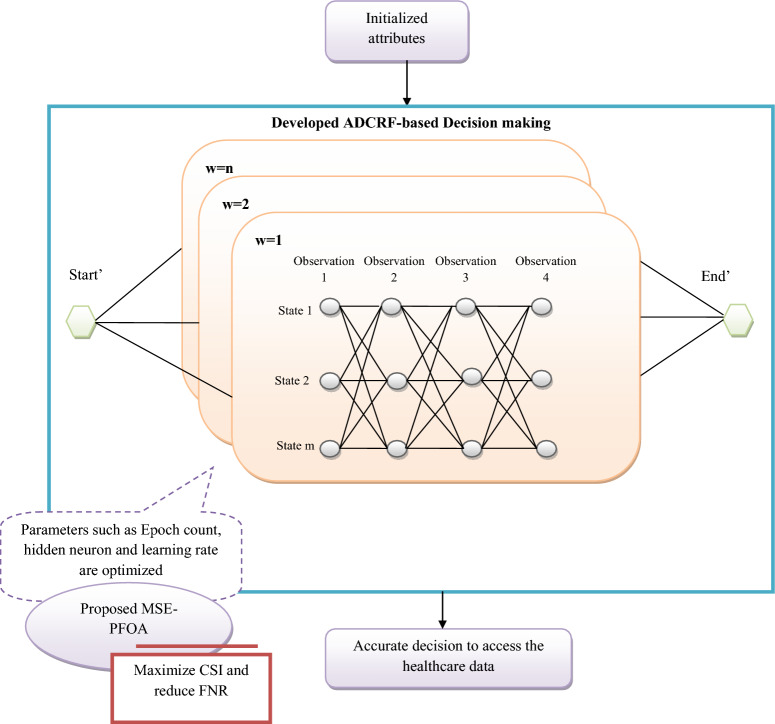
Designed ADCRF-based Decision Making Network.

Here, the true positive and negative values are expressed as $R_{p}$ and $R_{n}$ as well as the false positive and negative values are described as $G_{p}$ and $G_{n}$ in the above equation. The diagrammatic illustration of the designed ADCRF-based decision-making process is given in Fig. 3.

## Optimal Key-based Multi-Authority Attribute-Based Encryption with the support of Modified Escape Search-based Piranha Foraging Optimization Algorithm for secure data sharing and storage

### Introduced MES-PFOA for parameter and key optimization

*Purpose of MSE-PFOA:* In this designed secure data-sharing model, an effective tuning approach named MSE-PFOA is employed to perform parameter optimization during decision-making and optimal key selection for improving the overall effectiveness. The MSE-PFOA is employed to tune the hyperparameters of DCRF, including hidden neuron count, epochs and learning rate, which assist in improving CSI and minimizing FNR. At the encryption phase, MSE-PFOA is employed to select the keys optimally for executing encryption procedures. The optimal key selection process during encryption increases the key sensitivity, and the sensitive information of the patients is highly sensitive against malicious users.

*Design of MSE-PFOA:* The MSE-PFOA is an adaptive form of the standard PFOA technique. PFOA^34^ is a nature-inspired tuning approach that is designed based on the foraging habitat of piranhas. PFOA employ swarm intelligence techniques to prevent them from getting trapped in sub-optimal solutions, which makes them more applicable in addressing complex tuning issues. Moreover, it is more effective in real-time applications, where the condition varies over time. However, it struggles to offer effective performance for large-scale and complex tuning problems, resulting in sub-optimal solutions and high computational complexity.

*Novelty of MSE-PFOA: *Therefore, an adaptive form of the PFOA technique named MSE-PFOA is designed to address the limitations faced in the standard PFOA technique. The developed MES-PFOA is updated by an adaptive flag-based concept that regulates the direction of reverse escape search, which is significant for balancing exploration and exploitation phases and solves the issues of getting stuck in a local region. In order to eliminate several issues that take place in the classical PFOA, the flag ${\mathrm{T}}$ is utilized to modify the direction of the searching population during the reverse escape search strategy.

In the developed MES-PFOA, the flag attribute ${\mathrm{T}}$ is updated based on the fitness value of the piranhas, which includes best, worst and current fitness factors. This fitness-based flag updating process helps to explore better search regions with greater search probabilities. This attribute influences the local and global searching ability of piranhas, which helps to avoid premature convergence and provides efficient search behaviour in complex optimization issues. Due to this process, the slower convergence rate of traditional PFOA is effectively solved, and it modifies the movement direction of each piranha while searching for the reverse escape search. The flag ${\mathrm{T}}$ updating according to the fitness values is shown in Eq. (7).

7 $${\mathrm{T}} = \left\{ {\begin{array}{*{20}c} {1} & {\beta_{{3}} \le \frac{{Ft_{C} }}{{Ft_{W} + Ft_{B} }}} \\ { - 1} & {\beta_{{3}} > \frac{{Ft_{C} }}{{Ft_{W} + Ft_{B} }}} \\ \end{array} } \right.$$

Here, current, worst and best values associated with fitness are defined as $Ft_{C}$,$Ft_{W}$ and $Ft_{B}$. The arbitrary value within the limit $\left[ {0,1} \right]$ is expressed as $\beta_{{3}}$. The normalization of the best fitness position relative to all the members is helpful to direct the movement towards the best attribute solution.

In traditional PFOA, the flag ${\mathrm{T}}$ is calculated based on the random parameter, which lies between the values $\left[ {0,1} \right]$. The updated flag is provided in the position updating process of the piranha, which is given in Eq. (8).

8 $$y_{g} \left( {v + 1} \right) = \frac{1}{2}\left[ {e^{\vartheta 2} * y_{C} \left( r \right) - {\rm T} * y_{g} \left( r \right)} \right]$$

The updated position of the search agent is denoted as $y_{g} \left( {v + 1} \right)$, randomly selected agent to ensure the current interactions are given as $y_{C} \left( r \right)$, the uniformly distributed number within the range of $\left[ { - 1,1} \right]$ is represented as *ϑ 2*,$y_{g} \left( r \right)$ is the current position of the agents and ${\rm T}$ is the updated flag that helps to change the direction of the movement. Hence, the adoption of the flag ${\rm T}$ is helpful to improve the robustness and effectiveness of the solution searching process. The MES-PFOA gives more effective performance during both the decision-making across access control and encryption processes.

*Advantages of MES-PFOA:* The developed MES-PFOA operates as a closed-loop optimization process during training, where the objective function is considered for getting better candidate parameter configurations. The feedback loop is implemented based on the evaluation of candidates, performance assessment, fitness computation and adaptive position search mechanism. The hyperparameter vector corresponding to each piranha is used to train the DCRF model. Fitness value is computed based on the fitness function based on the fitness of the piranhas. The hyperparameter values are updated based on the position of Piranhas, which is repeated until it reaches the stopping criteria. This model evolves among the training cycles, which allows it to adapt to long-term environmental changes with higher stability.

The pseudocode of the introduced MES-PFOA is offered in Algorithm 1.

**Algorithm 1:**
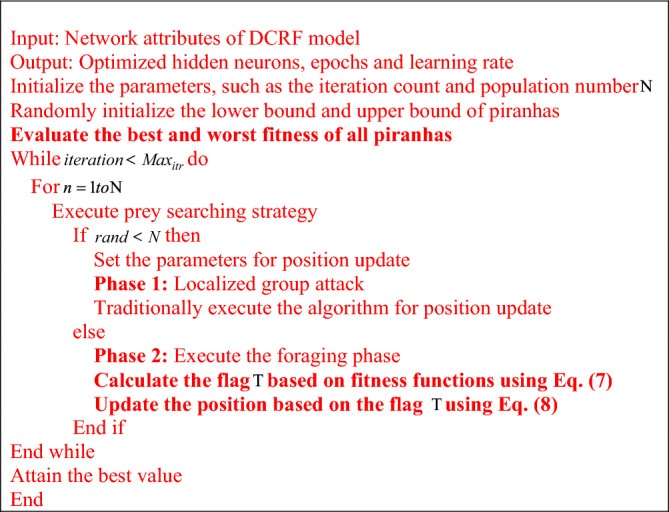
Implemented MSE-PFOA.

### Proposed data encryption scheme: O-MA-ABE

Data encryption is performed to encrypt the healthcare information of the user in order to preserve the data from third-party access. Here, the encryption process is executed by employing the designed O-MA-ABE technique. In O-MA-ABE, an optimal key is selected by the designed MES-PFOA optimization technique to improve the time and memory size. The MA-ABE^35^ is an extended form of the classical ABE, where multiple independent authorities are employed to handle diverse attribute sets, permitting precise access control to the encrypted information instead of depending on a single authority to handle all the attributes. Moreover, a user is required to attain the necessary attributes from each authority to decrypt the information, thereby enhancing the security of the healthcare information. Let us consider a message space *z* and class *v* for the tuple (Setup, Keyreq, KeyFGen, Keycomb, Encrypt, Decrypt) and the elements within the tuple are described below.

*Setup: *For the given security parameter *λ* and the set of authorities within the network *a*, the setup strategy creates a group of public parameters *pq* and a master secret key $ms_{k1} \ldots ms_{ka}$ for the authority as given in Eq. (9).

9 $$Setup\left( {1^{\lambda } ,1^{a} } \right) \to \left( {pq,ms_{k1} \ldots ms_{ka} } \right)$$

*Key request:* In the attribute string $c \in \left\{ {0,1} \right\}^{l}$, this phase presents a request string $req \in \left\{ {0,1} \right\}*$ and a receiver state *st* as shown in Eq. (10).

10 $$Keyreq_{pq} \left( c \right) \to \left( {req_{c} ,st} \right)$$

*KeyGen:* The key generation phase offers a key element $sk_{req\,j,n}$ for the master secret key $ms_{kj}$, a key request $req \in \left\{ {0,1} \right\}*$ and an attribute bit $n \in \left\{ {0,1} \right\}$ as shown in Eq. (11).

11 $$KeyGen_{pq} \left( {ms_{k} ,req,n} \right) \to sk_{req\,j,n}$$

*Key combine:* This phase presents a key $sk_{c}$ for the input recipient state *st* and a group of secret key elements $sk_{req,1,c1} , \ldots ,sk_{req,a,ca}$ as expressed in Eq. (12).

12 $$Keycomb_{pq} \left( {sk_{req,1,c1} , \ldots ,sk_{req,a,ca} } \right) \to sk_{c}$$

*Encrypt:* During encryption, a ciphertext *ct* is produced by considering the policy circuit $\varphi :\left\{ {0,1} \right\}^{a} \to \left\{ {0,1} \right\}$ within ${\mathrm{V}}$ and a message $\mu \in {\mathrm{Z}}$ as presented in Eq. (13).

13 $$Encrypt_{pq} \left( {\varphi ,\mu } \right) \to ct$$

*Decrypt:* Here, the decrypted message $\mu \in Z \cup \left\{ \bot \right\}$ is obtained by considering the decryption key $sk_{c}$ and the ciphertext *ct* as defined in Eq. (14).

14 $$Decrypt_{pq} \left( {sk_{c} ,ct} \right) \to \mu$$

**Fig. 4:**
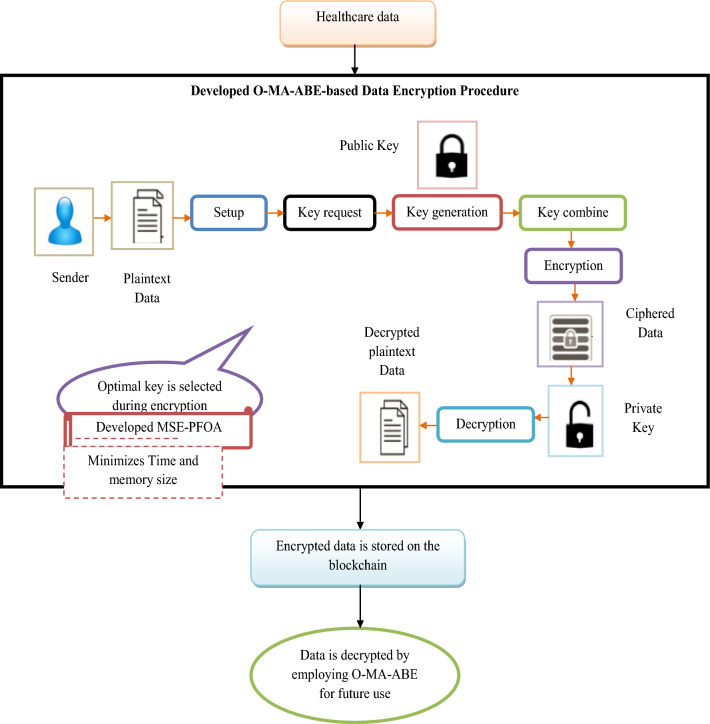
Designed O-MA-ABE-based Encryption Procedure.

After encryption, the patient’s healthcare information is preserved in blockchain. Here, the authenticated entities are permitted to decrypt the stored sensitive data for future use by employing O-MA-ABE. The architectural view of the introduced O-MA-ABE-based encryption procedure is presented in Fig. 4.

### Objective function of MES-PFOA-O-MA-ABE model

The key objective of the designed MES-PFOA-O-MA-ABE-based encryption model is to select an optimal key for the encryption procedure by employing the designed MES-PFOA optimization technique. This optimal key selection is performed to decrease the time as well as the memory size throughout the encryption process. The arithmetical form of the objective function is presented in Eq. (15).

15 $$Obj2 = \mathop {\arg \min }\limits_{{\left\{ {Op_{k}^{MA - ABE} } \right\}}} \left( {time + memorysize} \right)$$

Here, the optimal key selected by the implemented MES-PFOA within the range $\left[ {0,1} \right]$ is indicated as $Op_{k}^{MA - ABE}$.

- **Time:** It describes the time used by an encryption approach to execute both encryption and decryption procedures.
- **Memory size:** It defines the memory utilized by the encryption technique to preserve the encrypted as well as decrypted information during the implementation phase.

### Applying the developed framework in a real-world environment

In this research, a secure healthcare data sharing model is implemented by considering blockchain technology in the SDWBAN. The designed secure data sharing model in SDWBAN under the real-world condition uses the multi-layered procedures by considering the blockchain, decentralized storage and wearable storage in order to ensure overall security, patient privacy and data security.

Steps involved in implementing the developed framework under the real-world conditions:

- **Data Generation:** In order to collect various details from the patients, various sensors are used to monitor the patient’s health status and activities. In this phase, the medical sensors are used to obtain the physiological details like body temperature, heart beat rate, oxygen levels and blood sugar. Here, the WBAN controllers are employed to collect the patients health-related details, transfer them wirelessly through WiFi or Bluetooth, and compress them.
- **SDWBAN Controller**: In this phase, the smart devices, such as tablets and smartphones or other local edge servers, act as the controllers and optimize the data transmission.
- **Edge Pre-processing:** In this phase, the developed O-MA-ABE is employed to perform the encryption procedures and generate the hash functions.
- **Secure Data Transmission:** Due to the presence of enormous data samples, the raw data samples are securely stored in distributed storage like InterPlanetary File System (IPFS). Further, the sensitive information about the patients and the generated hash are transferred to the blockchain.
- **Smart Contract Execution:** To ensure immutability, access logs, smart contracts and data hashes are stored in the blockchain. Then, the smart contracts in the blockchain help to verify the stored data samples and update the mapping among the patient details.
- **Authorized Access:** Then, the authorized professionals use the private keys to access the data hash and restore the actual data from the IPFS. Here, the blockchain supports faster data transactions, controlled access, and ensures the privacy of data samples in various conditions.

Support of developed model in clinical environments:

- ***Managing Identity:*** In this phase, IoT devices, doctors and patients are registered with the developed framework. Then, the smart contract generates a unique blockchain address for managing the ownership of the data samples and user identity.
- ***Access Control for Patients:*** Here, the patients are allowed to utilize the smart contracts for allowing or rejecting the permissions for the doctors to access their medical information secured in IPFS.
- ***Legacy System Integration:*** In this phase, the standardized Application Programming Interface (API) are employed to execute the communication process in the blockchain over the prior Electronic Health Records (EHR) and hospital information system.
- ***Validating Data:*** Before the data samples are forwarded to the blockchain, a deep validation is executed to verify the data tampering and also verify the signed data samples.

Thus, the developed model is capable of deploying under real-world conditions such as remote patient monitoring, hospitals and telemedicine platforms.

### Handling attacks and cyberthreats

The developed secure data sharing model is capable of protecting the system from various cyber threats that harm IoT-based healthcare environments. Different attacks prevented by the developed model are data tampering attacks, unauthorized attacks, Man-In-The-Middle (MITM) and replay attacks. These attacks may affect the overall integrity and confidentiality of the sensitive medical information that takes place in the SDWBAN system. Thus, to tackle these issues, the developed framework has included the permissioned blockchain for secure data storage and an O-MA-ABE-based encryption scheme for secure access control. Thus, the proposed technique is capable of securing the sensitive patients details from cyber threats and attacks.

*Prevention of Cyber Attacks using Developed Model.* In the designed secure data sharing model, the ADCRF works as the major controller for SDWBAN, which is capable of identifying the intrusions. Moreover, the novel optimization scheme MES-PFOA is employed for tuning the hyperparameters and accomplished higher sensitivity over different attacks. This process is capable of detecting the False Data Injection (FDI) attacks by recognizing the deviations in the sensor patterns. Moreover, the designed encryption scheme O-MA-ABE is efficient, ensuring the node security. In this phase, the attackers are unable to decrypt the sensitive data as specific attributes are available for access, and they are verified through various authorities. The developed O-MA-ABE is efficient in mitigating the brute-force decryption attacks and collusion attacks. In order to maintain higher data integrity, a secure blockchain is used, which aids in preventing the network from MITM attacks. Once the sensitive medical information is hashed and recorded in the blockchain, the attackers are unable to perform any modifications as quick detection is performed through consensus. *Quantum Vulnerability.* In addition, the developed technique is efficient in tackling the quantum vulnerability issues with the help of O-MA-ABE. In this phase, decentralized trust is maintained over the authorities, and higher data integrity is maintained. Moreover, the immutable hashing on the blockchain supports all the data samples from the attackers. Here, the developed O-MA-ABE, along with ADCRF and blockchain, aids in protecting the network from quantum vulnerability issues.

*Testing the Developed Model over Cyber Attacks.* The developed technique is capable of handling various cyber attacks, and its details are listed as follows. In the FDI attack, malicious nodes are programmed to provide false medical alerts to the user. In this case, the developed ADCRF-MES-PFOA is used to validate the temporal dependencies in the data samples. So, the injected data didn’t able to follow the physiological patterns of the patients, and it is termed as anomalous. Next, in the unauthorized access and collusion attacks, several unauthorized entities are used to fuse the attributes and make different guesses at the description keys related to the sensitive health records. In order to rectify these issues, an efficient optimization scheme O-MA-ABE is employed to verify the attributes over various independent authorities. Here, decryption failure takes place for all the unauthorized attempts with zero data breach. Further, at the MITM attack, the attackers may intercept the data packets at the transmission phase and may perform the modification of the medical timestamps. In this phase, the system is contrasted over the hash stored in the blockchain and the modified packet. Here, the developed technique supports identifying the tampered data packets quickly and eliminating them before they reach the database.

## Results and discussions

### Experimental setup

MATLAB 2020a was employed for the execution of the suggested secure data-sharing system. Here, the implementation platform was designed by considering the population and the highest iteration count as 10 and 50. In addition, the chromosome length during decision-making and encryption procedures was considered to be 3 and 16.

Furthermore, traditional tuning approaches include Mud Ring Algorithm (MRA)^36^, Flamingo Search Algorithm (FSA)^37^, Addax Optimization Algorithm (AOA)^38^, and PFOA^38^. Encryption approaches like Elliptic Curve Cryptography (ECC)^39^ and Rivest–Shamir–Adleman (RSA)^40^, Data Encryption Standard (DES)^41^ and MA-ABE^35^ and classifiers such as RF^1^, Deep Neural Network (DNN)^42^, Long Short-Term Memory (LSTM)^24^ and DCRF^33^ were employed to evaluate the competence of the suggested system was estimated by analyzing the performance of the suggested framework. Furthermore, existing blockchain models like Advanced Hierarchical Identity-based Security Mechanism by Blockchain (AHISM-B)^43^, Blockchain Technology-Featured Air-Cracking Tool (BTFAT)^44^, Blockchain-enabled Deep Quantum Computing Model (B-DQCM)^45^ and Blockchain User Datagram Transport Protocol with Reinforcement Projection Regression (BUDTP-RPR)^46^ were employed to estimate the performance of the blockchain model. Recent data sharing models used for the analysis were DTAC-TL-QM^29^, SCCE-DS^41^, BFL-hIoT^31^ and PPFL-ICP^32^.

Accessible code for the developed model has been provided in the link https://github.com/sumanthvmani/Decision-Making-using-ADCRF-with-Encryption/tree/main.

Various parameters considered in the research work are listed in Table 2.

**Table 2:** Details of Experimental Validation Parameters.

Category	Parameters	Configuration	
Dataset	Name of the Dataset	Diabetes Dataset	
	Attributes	Skin thickness, weight, Blood pressure, Glucose level, Insulin, Age,	
	Source	National Institute of Diabetes and Kidney Diseases (Kaggle)	
	Training	75% Samples	
	Testing	25% Samples	
Hardware Configuration	Processor	Intel i3	
	Storage	500 GB	
	RAM	8 GB	
	Operating System	Windows 11	
Implementation Platform	Programming Tools	Python	
	Python Environment	PyCharm, Anaconda (Python 3.11)	
Libraries	ML & Visualization Libraries	Matplotlib, TensorFlow, OpenCV, Keras, NumPy, TFlearn, PrettyTable	
Optimization Parameters	Chromosome Length	3 for decision making	16 for encryption
	Maximum Iterations	50	
	Population Size	10	
Blockchain Configuration	Blockchain Type	Permissioned Blockchain	
	Consensus Algorithm	Practical Byzantine Fault Tolerance (PBFT)	
	Block Creation Interval	2 s	
	Block Size	1 MB	
	Transactions per Block	200	
SDWBAN Network Setup	Sensor Nodes	10–25 wearable biomedical sensors	
	Controller	SDN Edge Controller	
	Communication Protocol	IEEE 802.15.6	
Compared Models	Encryption Models	ECC, RSA, DES, MA-ABE	
	Decision Models	RF, DNN, LSTM, DCRF	
	Optimization Methods	MRA, FSA, AOA, PFOA	
	Blockchain Models	BUDTP-RPR, B-DQCM, BTFAT, AHISM-B	
	Recent data sharing models	DTAC-TL-QM, SCCE-DS, BFL-hIoT, PPFL-ICP	

### Evaluation measures

The designed secure data-sharing approach’s competence is evaluated based on the listed measures.

- Computational time is the time employed by the developed encryption strategy, which utilizes more time for processing.
- Decryption time is the time consumed by the approach for decrypting the encrypted data to plaintext.
- Encryption time is the duration employed by the cryptography approach to implement encryption procedures.
- CSI is expressed in Eq. (5)
- FNR is derived using Eq. (6).
- False Positive Rate (FPR) is attained by Eq. (16).
  16 $$FPR = \frac{{G_{p} }}{{G_{p} + R_{n} }}$$
- Matthews Correlation Coefficient (MCC) is defined by Eq. (17).
  17 $$MCC = \frac{{\left( {\left( {R_{p} * R_{n} } \right) - \left( {G_{p} * G_{n} } \right)} \right)}}{{\sqrt {\left( {R_{p} + G_{p} } \right) * \left( {R_{p} + G_{n} } \right) * \left( {R_{n} + G_{p} } \right) * \left( {R_{n} + G_{n} } \right)} }}$$
- Precision is expressed using Eq. (18).
  18 $$\Pr ecision = \frac{{R_{p} }}{{\left( {R_{p} + G_{p} } \right)}}$$
- (i)Sensitivity is described by Eq. (19).
  19 $$Sensitivity = \frac{{R_{p} }}{{\left( {R_{p} + G_{n} } \right)}}$$
- Accuracy is defined using Eq. (20).
  20 $$Acc = \frac{{R_{p} + R_{n} }}{{R_{p} + R_{n} + G_{p} + G_{n} }}$$

The major objective of the designed secure data sharing model is to fulfill the fitness function provided in Eq. (4) for accurate decision making and Eq. (15) for cryptography. In order to improve the clarity, different validations are performed by considering the core objectives. Here, the efficiency of the developed work is analyzed by considering the primary measures such as encryption time, FNR, memory size and CSI, which work as key elements to verify the overall system efficiency. The remaining metrics are retained as supporting evaluation measures to provide a comprehensive analysis of system reliability and security.

### Activation function-based decision-making performance analysis

**Fig. 5:**
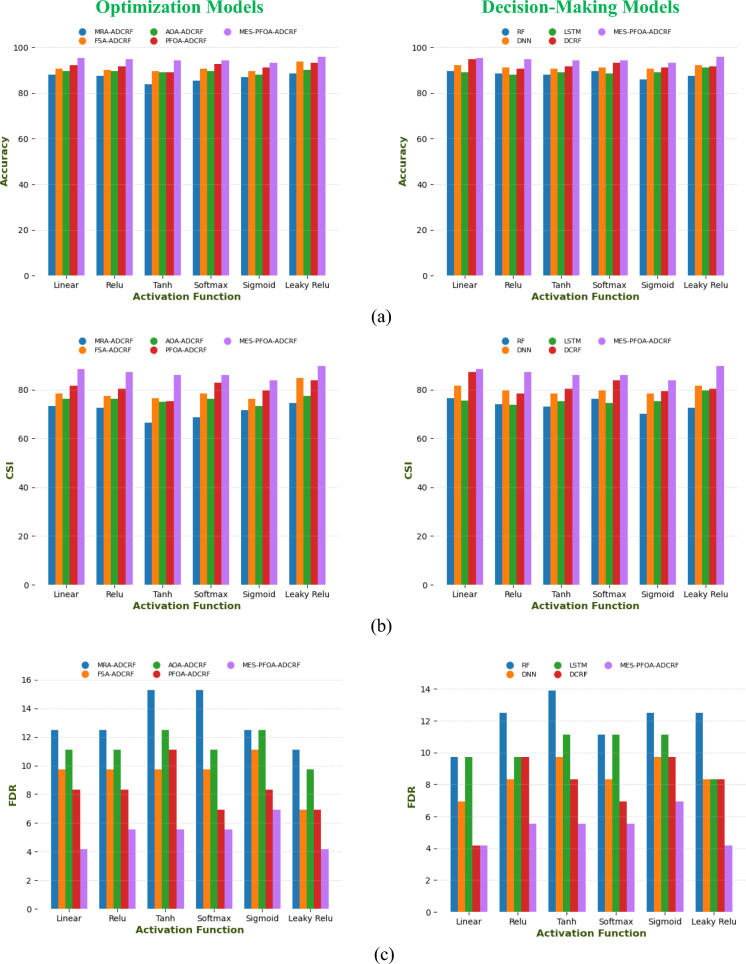

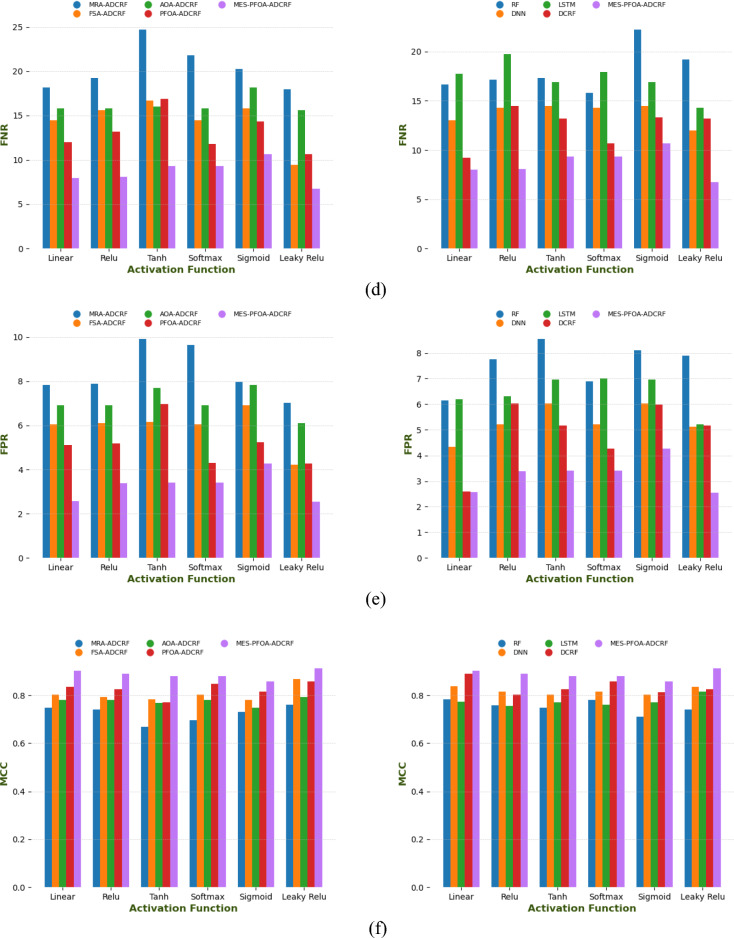

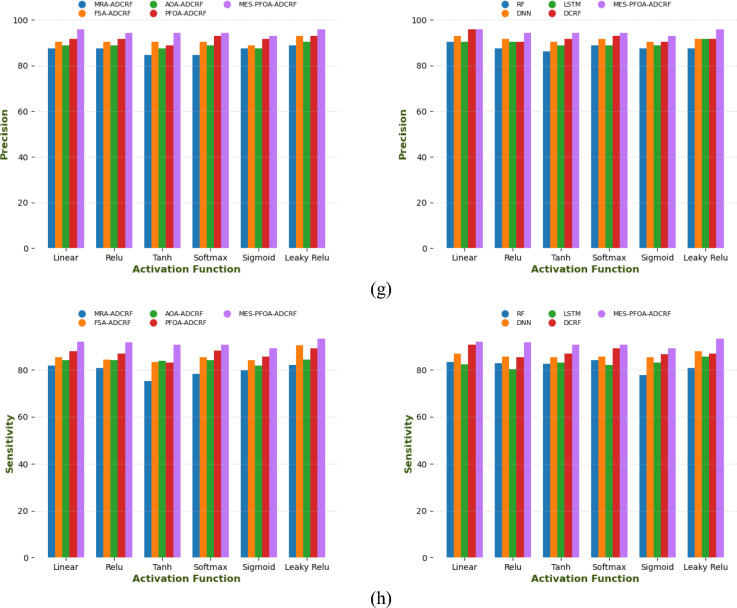
Decision Making Performance Evaluation regarding **a** Accuracy, **b** CSI, **c** FDR, **d** FNR, **e** FPR, **f** MCC, **g** Precision, and **h** Sensitivity.

The performance of the designed ADCRF-based decision-making model is evaluated by adjusting the activation function, and the resultant values are analyzed with traditional tuning approaches and several security approaches. The evaluation outcomes attained by the suggested system are presented in Fig. 5. Employing diverse activation functions permits the model to understand the complicated connections within the given input. From Fig. 5a, the accuracy of the designed system is 9.41%, 4.49%, 5.08%, and 3.33% more advanced than traditional optimization approaches like MRA-ADCRF, FSA-ADCRF, AOA-ADCRF and PFOA-ADCRF as well as 9.19%, 5.55%, 10.46% and 6.14% superior to standard models like RF, DNN, LSTM and DCRF, while taking the ReLU function. The results clearly state that the RELU activation function attains more effective performance when compared to other functions, because it avoids saturation for positive inputs. Moreover, the gradients are stable and large across layers. Moreover, the vanishing gradient effects are low when compared to other activation functions like tanh and sigmoid. The ADCRF model captures the complex relationships against the system attributes, such as user behaviour and SDN network characteristics. While analyzing with other measures such as FDR, FNR and FPR proves the reduced error rate while making the decisions related to access control. Therefore, according to the attained outcomes, the designed ADCRF system is more effective in determining accurate decisions to ensure secure data sharing within the SDWBAN. Hence, the results proved the secure performance of our model in healthcare systems.

### Performance analysis for the designed blockchain model

**Fig. 6:**
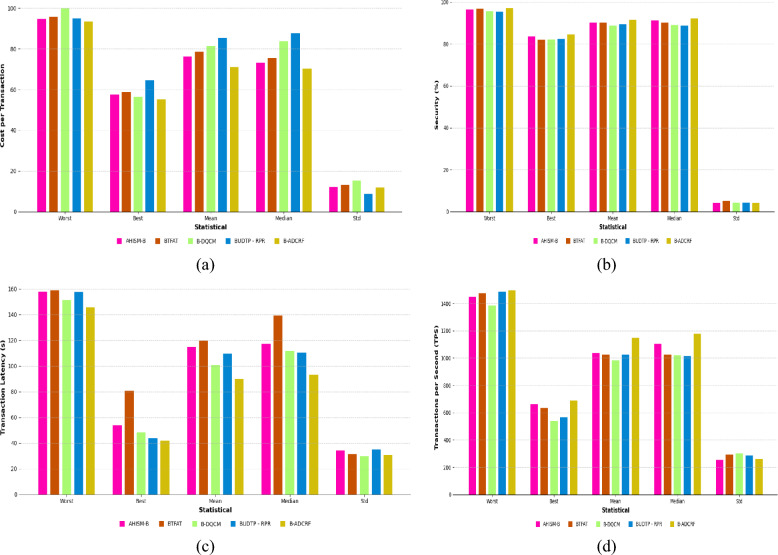
Performance analysis for the designed Blockchain model regarding **a** Cost per Transaction, **b** Security, **c** Transaction latency, and **d** Transactions per second.”

The performance of the introduced blockchain-based ADCRF for decision making is analyzed with existing blockchain models, and the obtained results are presented in Fig. 6. The key intent of this model is to ensure the security of the patient’s personal information that is preserved on the blockchain. From Fig. 6b, the security of the developed blockchain-aided ADCRF model is 1.11%, 1.67%, 3.40%, and 2.24% more effective than existing models like AHISM-B, BTFAT, B-DQCM and BUDTP-RPR, while considering the mean performance. The latency analysis illustrates the reduced delay of the proposed blockchain model with an encryption mechanism, and hence, it is highly suitable for real-time healthcare applications. The results clearly illustrate that the blockchain, with its decision making ability regarding access control, helps to protect sensitive data from unauthorized access. This evaluation indicates that the suggested ADCRF-aided decision-making model is more effective in guaranteeing the privacy of the data preserved on the blockchain from unauthenticated access. The adoption of an encryption module highly preserves the patient data with reduced latency and cost.

### K-fold-aided decision making performance evaluation

The overall decision-making performance of the suggested ADCRF system across classical techniques is tabulated in Tables 3 and 4. Employing diverse K-fold values permits the developed model to evaluate its efficiency in various experimental scenarios. Moreover, this evaluation presents higher robustness and shows how well the suggested system processes unknown information. This outcome assists in determining the generalization ability of the system. According to the attained results in Table 4, the CSI value of the suggested MES-PFOA-ADCRF exceeds classical models like RF, DNN, LSTM and DCRF by 15.00%, 6.72%, 10.28% and 1.49%, while considering the k-fold as 3. Therefore, it is clear that the designed model is more effective in decision-making in dynamic conditions than conventional models.

**Table 3:** K-fold-aided Decision Making Performance Evaluation over Traditional Tuning Approaches.

Performance Measures	MRA-ADCRF^36^	FSA-ADCRF^37^	AOA-ADCRF^38^	PFOA-ADCRF^34^	MES-PFOA-ADCRF
When k-fold is 1					
Accuracy	88.311688	88.961039	87.012987	90.909091	92.207792
Specificity	92.134831	92.222222	90.10989	94.382022	94.505495
FPR	7.8651685	7.7777778	9.8901099	5.6179775	5.4945055
FNR	16.923077	15.625	17.460317	13.846154	11.111111
FDR	11.47541	11.47541	14.754098	8.1967213	8.1967213
F1-score	85.714286	86.4	83.870968	88.888889	90.322581
MCC	0.7595057	0.7718233	0.7303275	0.8132698	0.8383423
CSI	75	76.056338	72.222222	80	82.352941
When k-fold is 2					
Accuracy	90.25974	92.207792	88.311688	92.207792	94.155844
Specificity	93.333333	94.505495	92.134831	94.505495	95.652174
FPR	6.6666667	5.4945055	7.8651685	5.4945055	4.3478261
FNR	14.0625	11.111111	16.923077	11.111111	8.0645161
FDR	9.8360656	8.1967213	11.47541	8.1967213	6.557377
F1-score	88	90.322581	85.714286	90.322581	92.682927
MCC	0.7987636	0.8383423	0.7595057	0.8383423	0.8782664
CSI	78.571429	82.352941	75	82.352941	86.363636
When k-fold is 3					
Accuracy	88.961039	90.25974	88.961039	90.25974	93.506494
Specificity	93.181818	93.333333	93.181818	93.333333	95.604396
FPR	6.8181818	6.6666667	6.8181818	6.6666667	4.3956044
FNR	16.666667	14.0625	16.666667	14.0625	9.5238095
FDR	9.8360656	9.8360656	9.8360656	9.8360656	6.557377
F1-score	86.614173	88	86.614173	88	91.935484
MCC	0.7742021	0.7987636	0.7742021	0.7987636	0.865346
CSI	76.388889	78.571429	76.388889	78.571429	85.074627
When k-fold is 4					
Accuracy	86.363636	90.909091	86.363636	88.961039	90.25974
Specificity	90.909091	94.382022	90.909091	93.181818	93.333333
FPR	9.0909091	5.6179775	9.0909091	6.8181818	6.6666667
FNR	19.69697	13.846154	19.69697	16.666667	14.0625
FDR	13.114754	8.1967213	13.114754	9.8360656	9.8360656
F1-score	83.464567	88.888889	83.464567	86.614173	88
MCC	0.7205445	0.8132698	0.7205445	0.7742021	0.7987636
CSI	71.621622	80	71.621622	76.388889	78.571429
When the k-fold is 5					
Accuracy	87.012987	90.25974	88.311688	93.506494	94.805195
Specificity	91.011236	93.333333	92.134831	95.604396	96.703297
FPR	8.988764	6.6666667	7.8651685	4.3956044	3.2967033
FNR	18.461538	14.0625	16.923077	9.5238095	7.9365079
FDR	13.114754	9.8360656	11.47541	6.557377	4.9180328
F1-score	84.126984	88	85.714286	91.935484	93.548387
MCC	0.7326236	0.7987636	0.7595057	0.865346	0.8923497
CSI	72.60274	78.571429	75	85.074627	87.878788

**Table 4:** K-fold-aided Decision Making Performance Evaluation over Classical Models.

Performance Measures	RF^1^	DNN^42^	LSTM^47^	DCRF^33^	MES-PFOA-ADCRF
When k-fold is 1					
Accuracy	88.311688	88.311688	90.909091	91.558442	92.207792
Specificity	91.208791	91.208791	93.406593	94.444444	94.505495
FPR	8.7912088	8.7912088	6.5934066	5.5555556	5.4945055
FNR	15.873016	15.873016	12.698413	12.5	11.111111
FDR	13.114754	13.114754	9.8360656	8.1967213	8.1967213
F1-score	85.483871	85.483871	88.709677	89.6	90.322581
MCC	0.7573312	0.7573312	0.8113386	0.825704	0.8383423
CSI	74.647887	74.647887	79.710145	81.15942	82.352941
When k-fold is 2					
Accuracy	90.909091	90.909091	92.207792	91.558442	94.155844
Specificity	93.406593	93.406593	94.505495	94.444444	95.652174
FPR	6.5934066	6.5934066	5.4945055	5.5555556	4.3478261
FNR	12.698413	12.698413	11.111111	12.5	8.0645161
FDR	9.8360656	9.8360656	8.1967213	8.1967213	6.557377
F1-score	88.709677	88.709677	90.322581	89.6	92.682927
MCC	0.8113386	0.8113386	0.8383423	0.825704	0.8782664
CSI	79.710145	79.710145	82.352941	81.15942	86.363636
When k-fold is 3					
Accuracy	87.662338	90.909091	89.61039	92.857143	93.506494
Specificity	92.045455	93.406593	92.307692	95.555556	95.604396
FPR	7.9545455	6.5934066	7.6923077	4.4444444	4.3956044
FNR	18.181818	12.698413	14.285714	10.9375	9.5238095
FDR	11.47541	9.8360656	11.47541	6.557377	6.557377
F1-score	85.03937	88.709677	87.096774	91.2	91.935484
MCC	0.7473733	0.8113386	0.7843349	0.8526443	0.865346
CSI	73.972603	79.710145	77.142857	83.823529	85.074627
When k-fold is 4					
Accuracy	87.012987	87.012987	90.25974	90.25974	90.25974
Specificity	91.011236	91.011236	93.333333	93.333333	93.333333
FPR	8.988764	8.988764	6.6666667	6.6666667	6.6666667
FNR	18.461538	18.461538	14.0625	14.0625	14.0625
FDR	13.114754	13.114754	9.8360656	9.8360656	9.8360656
F1-score	84.126984	84.126984	88	88	88
MCC	0.7326236	0.7326236	0.7987636	0.7987636	0.7987636
CSI	72.60274	72.60274	78.571429	78.571429	78.571429
When the k-fold is 5					
Accuracy	90.909091	92.857143	90.25974	92.857143	94.805195
Specificity	93.406593	94.565217	93.333333	95.555556	96.703297
FPR	6.5934066	5.4347826	6.6666667	4.4444444	3.2967033
FNR	12.698413	9.6774194	14.0625	10.9375	7.9365079
FDR	9.8360656	8.1967213	9.8360656	6.557377	4.9180328
F1-score	88.709677	91.056911	88	91.2	93.548387
MCC	0.8113386	0.8511942	0.7987636	0.8526443	0.8923497
CSI	79.710145	83.58209	78.571429	83.823529	87.878788

### Convergence-based performance analysis

**Fig. 7:**
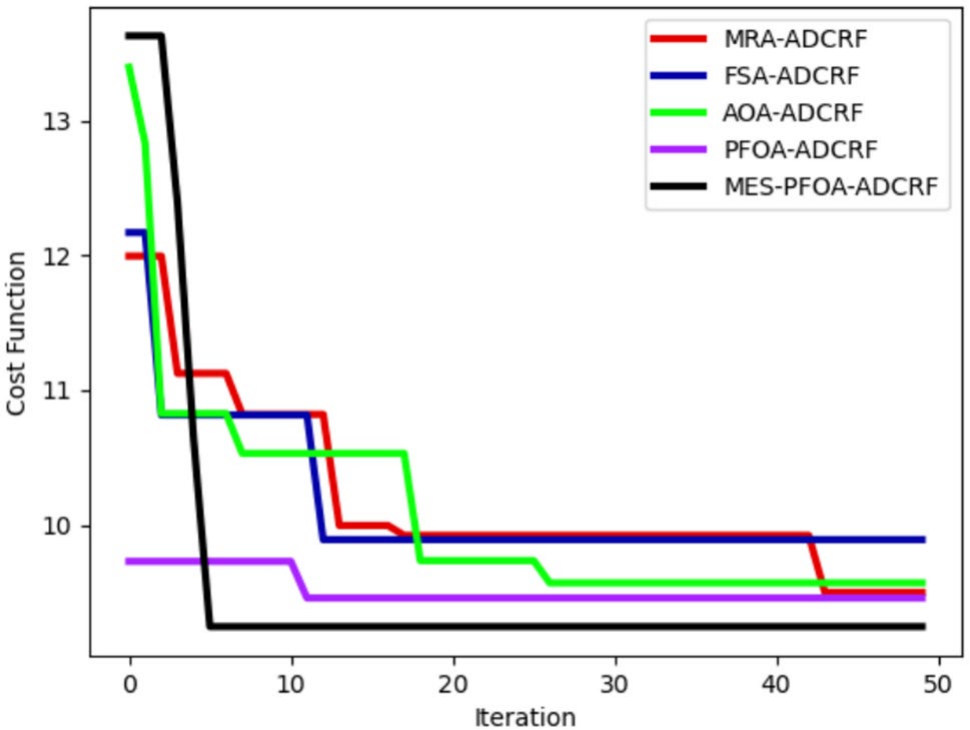
Convergence-based Performance Analysis.

The convergence graph attained by the suggested decision-making system is offered in Fig. 7. This evaluation is significant for estimating the learning ability of the designed approach at the training phase. In addition, this validation enables the model to determine the overfitting and underfitting issues within the system that assist in suggesting effective measures to address these problems. Here, the overall performances are verified by varying the iteration counts. From the given graph, the suggested MES-PFOA-ADCRF-based decision-making model has attained a greater convergence rate than conventional approaches, including MRA-ADCRF, FSA-ADCRF, AOA-ADCRF, and PFOA-ADCRF. This evaluation reveals that the effectiveness of the suggested approach during decision-making is higher for guaranteeing the privacy of the data within the network.

### ROC-based decision-making efficiency analysis

**Fig. 8:**
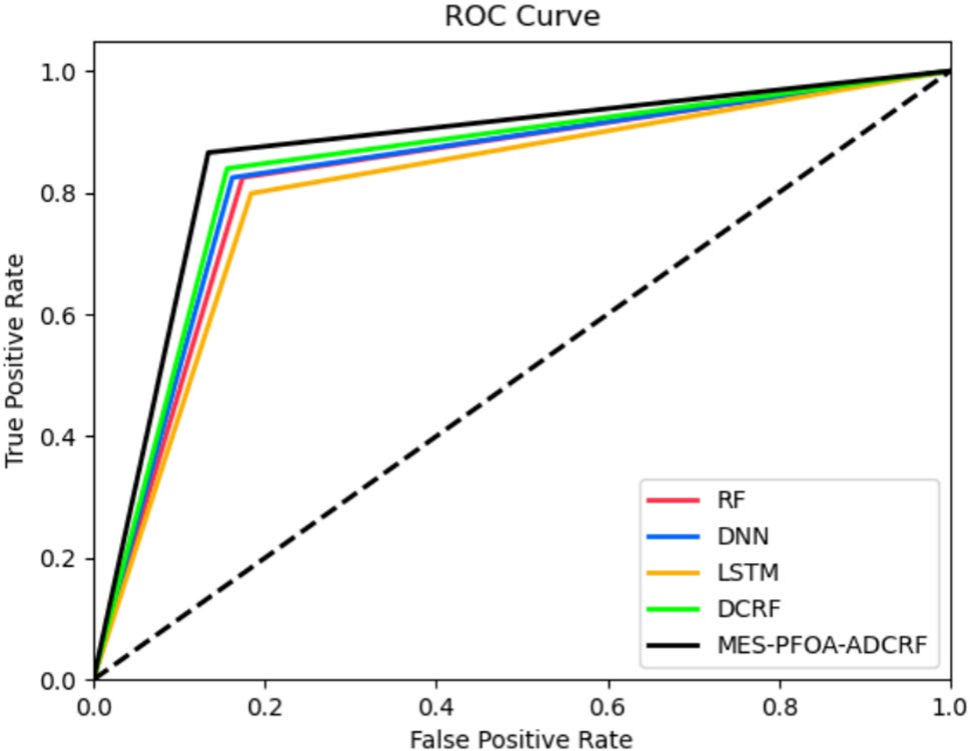
ROC-based Decision-Making Efficiency Analysis.

The ROC curve of the introduced decision-making framework is given in Fig. 8. Here, the ROC graph is attained by analyzing the competence of the introduced system with existing decision-making models. The ROC curve offers a complete review based on the efficiency of the model with respect to optimal threshold values. Moreover, it analyzes the overall competence of the system based on negative and positive classes. In the ROC graph, the AUC value is taken as an effective measure to estimate the competence of the system. In the validation, the model with a high AUC value is considered as the best model to address the overfitting issues. According to the attained results, the effectiveness of the suggested MES-PFOA-ADCRF model has outperformed the traditional approaches by offering accurate decisions based on user access.

### Encryption efficiency estimation with standard models

**Fig. 9:**
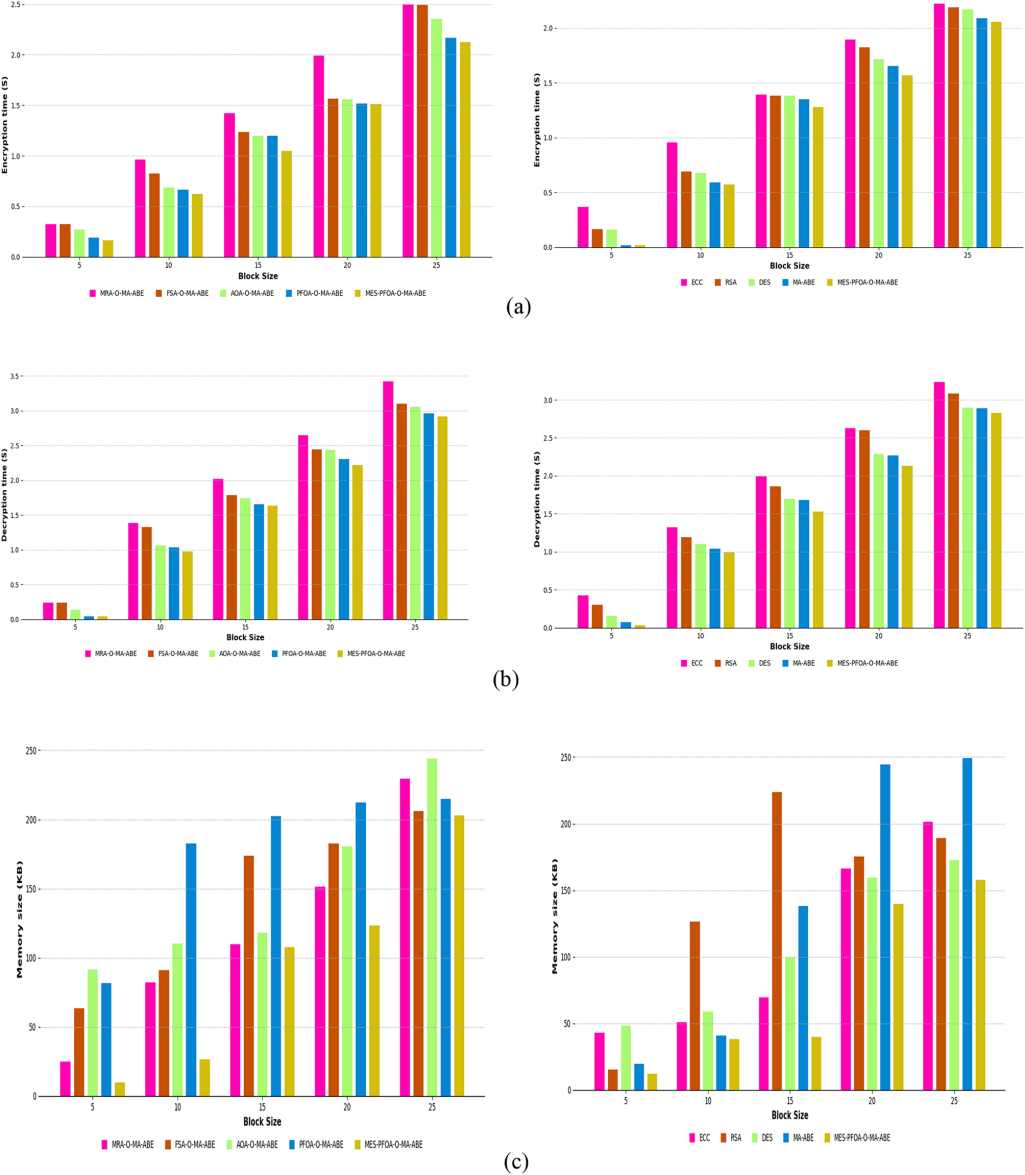

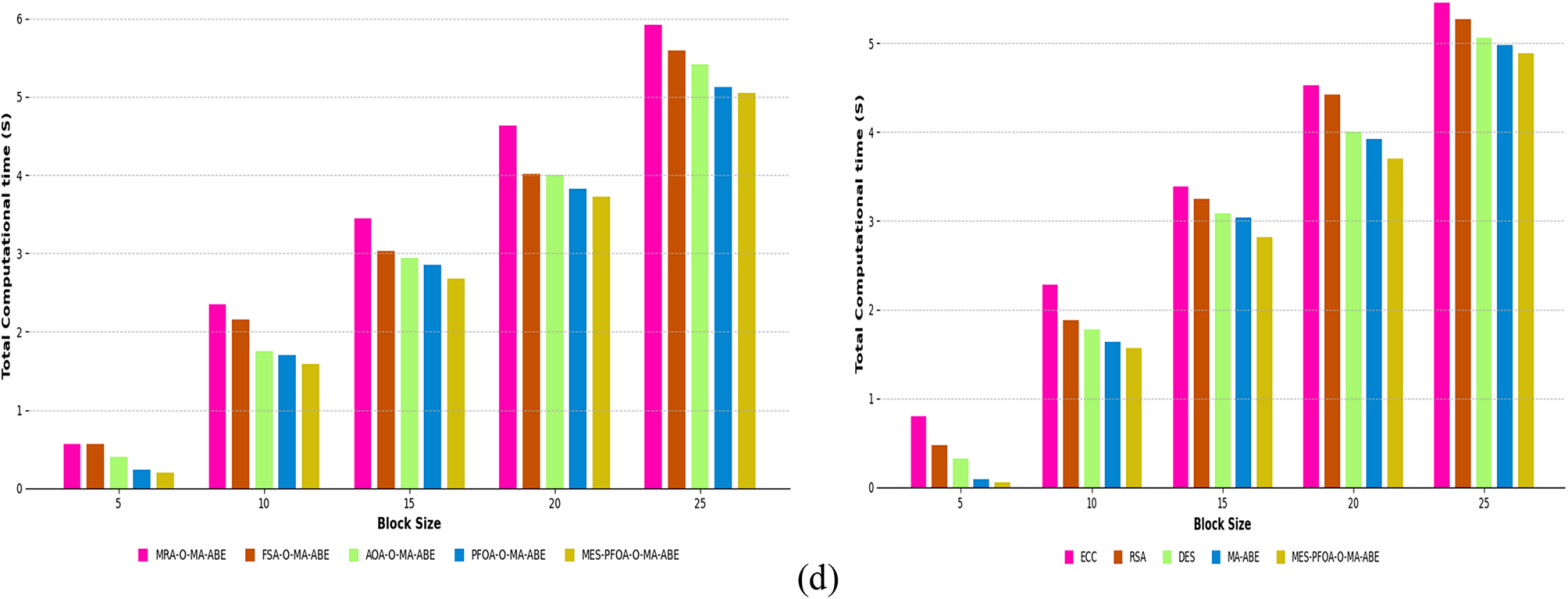
Encryption Performance Evaluation regarding **a** Encryption time, **b** Decryption time, **c** Memory size, and **d** Total computational time”.

**Fig. 10:**
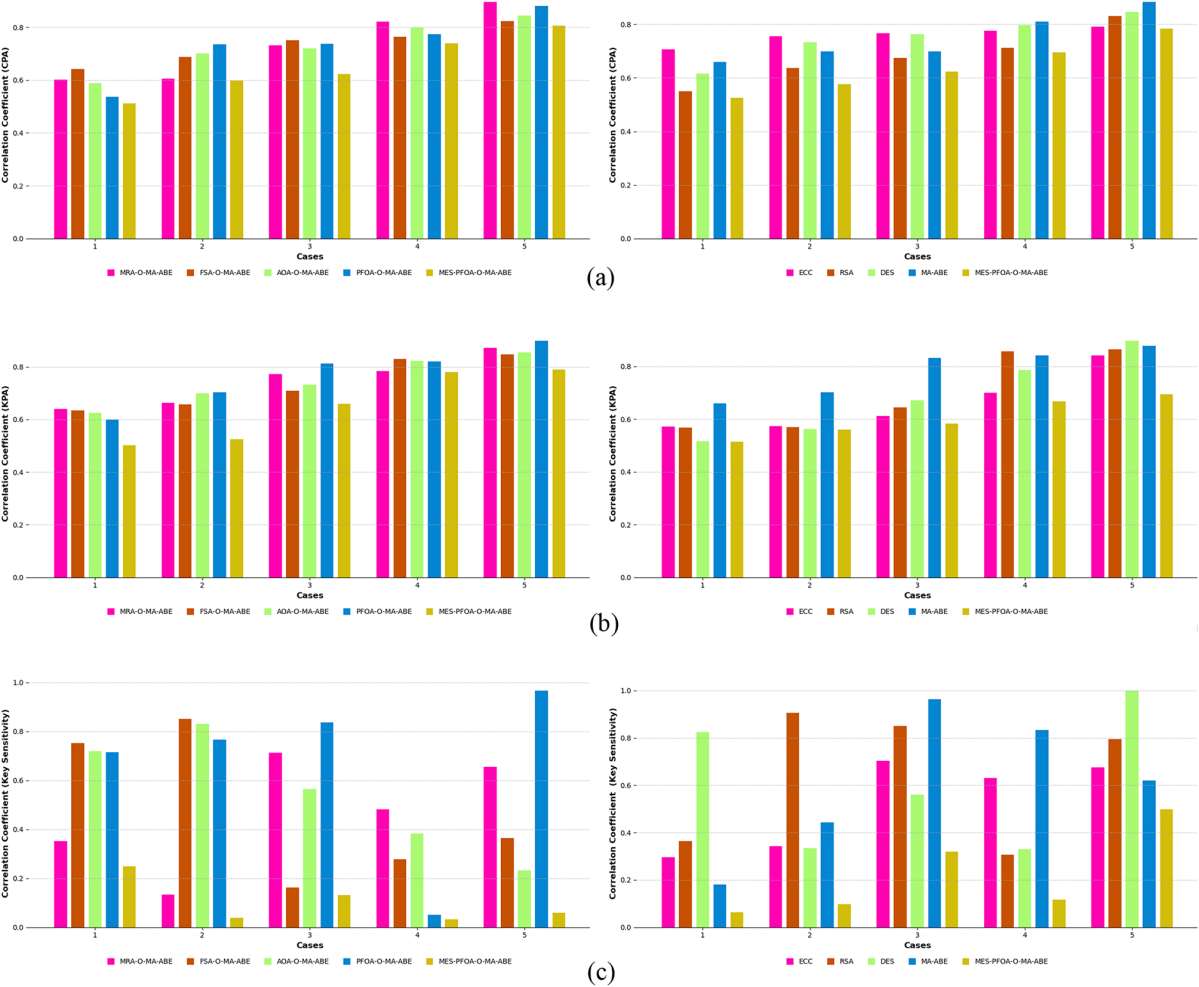
Attack-based Efficiency Analysis regarding **a** CPA, **b** KPA, and **c** Key Sensitivity.

The efficiency of the designed MES-PFOA-O-MA-ABE-based cryptographic approach during the encryption and decryption procedures across classical models is evaluated by varying the block sizes, and resultant graphs are provided in Fig. 9. Here, the efficiency of the designed MES-PFOA-O-MA-ABE is estimated by considering the metrics including encryption and decryption time, total computation time and memory size. Varying the block size during encryption performance evaluation presents a better understanding, which enhances the privacy of preserved data in the network. While considering Fig. 10a, the encryption performance of the proposed technique is 40.40%, 15.71%, 14.49%, and 1.66% more advanced than classical encryption procedures like ECC, RSA, DES, and MA-ABE. Additionally, in the decryption time analysis, the developed MES-PFOA-O-MA-ABE has achieved 34.34%, 18.75%, 7.14%, and 5.79% effective performance than MRA-O-MA-ABE, FSA-O-MA-ABE, AOA-O-MA-ABE, and PFOA-O-MA-ABE, for the block size 10. Hence, this evaluation proved that the designed encryption technique MES-PFOA-O-MA-ABE has offered higher efficiency in the encryption procedures. This effective encryption performance is attained over the medical data because our encryption method uses sensitive parameters and intermediate representations only. The minimized number of encrypted operations avoids the heavy overhead associated with end-to-end encryption methods. It provides block-level encryption, and it uses symmetric key operations for the encryption process, which helps to minimize the latency.

### Attack analysis on developed MES-PFOA-O-MA-ABE

The performance of the designed MES-PFOA-O-MA-ABE is estimated by performing a CPA and KPA analysis as presented in Fig. 10. Attack-based efficiency evaluation assists the system in monitoring how the competence of the encryption approach is influenced by attack, which is significant to design effective measures to overcome attacks. In this evaluation, the CPA attack evaluation presented in Fig. 10a is considered, and the simulation results revealed that the competence of developed MES-PFOA-O-MA-ABE exceeds the traditional approaches like ECC, RSA, DES, and MA-ABE by 25.64%, 6.45%, 22.66% and 17.14%, for the case count 2. Thus, the final outcomes displayed that the developed MES-PFOA-O-MA-ABE is efficient in obtaining better outcomes over several attacks and ensures the overall security under the dynamic conditions.

### Statistical analysis measures–based performance evaluation

Statistical reports attained by the designed decision-making and encryption techniques are given in Table 5. This evaluation is essential to determine the effectiveness of the introduced secure data-sharing model for SDWBAN. Here, a few statistical measures like best, mean, standard deviation, worst and median values are taken for evaluation procedures. From Table 5, the developed MES-PFOA-ADCRF-based decision-making technique is 13.61%, 11.98%, 1.73%, and 40.03% more effective than MRA-ADCRF, FSA-ADCRF, AOA-ADCRF, and PFOA-ADCRF, while taking the worst measure. Moreover, the developed MES-PFOA-O-MA-ABE-aided encryption approach accomplished higher efficiency as 6.72%, 3.96%, 3.76%, and 4.93%, superior to MRA-O-MA-ABE, FSA-O-MA-ABE, AOA-O-MA-ABE, and PFOA-O-MA-ABE, while considering the mean value of block size 10. Therefore, the resultant values proved that the introduced data-sharing model is effective in performing secure data transmission on the SDWBAN model. The experimental averaging and variance analysis with ten independent runs has been conducted to ensure the randomness and stability of the presented approach. Moreover, the reduction of precision results in terms of rounded values to ensure the accuracy of the final results. Finally, the statistical significance testing in terms of t-value and p-values has been provided in these results.

**Table 5:** Statistical measures–based Performance Analysis for Decision-making.

Performance Measures	MRA-ADCRF^36^	FSA-ADCRF^37^	AOA-ADCRF^38^	PFOA-ADCRF^34^	MES-PFOA-ADCRF
Best	9.5003422	9.8920448	9.5712508	9.4593725	9.2473848
Mean	10.198266	10.168034	10.076072	9.519163	9.6005731
Median	9.9239117	9.8920448	9.7369344	9.4593725	9.2473848
Worst	11.994081	12.168866	13.393952	9.7311474	13.626977
Standard deviation	0.6474458	0.5496879	0.7837837	0.1125817	1.1230369

Experimental Averaging and Variance Analysis (10 Independent Runs)					
Models	Accuracy (Mean ± Std)	F1-Score (Mean ± Std)	MCC (Mean ± Std)		
RF	0.883 ± 0.012	0.855 ± 0.015	0.757 ± 0.018		
DNN	0.889 ± 0.014	0.862 ± 0.017	0.764 ± 0.019		
LSTM	0.905 ± 0.011	0.887 ± 0.013	0.812 ± 0.014		
Static DCRF	0.914 ± 0.009	0.896 ± 0.011	0.826 ± 0.012		
MES-PFOA-ADCRF	0.931 ± 0.006	0.914 ± 0.008**	0.857 ± 0.009		

Reduction of Apparent Precision (Rounded Results)					
Models	Accuracy	F1-Score	MCC		
RF	0.883	0.855	0.757		
DNN	0.889	0.862	0.764		
LSTM	0.905	0.887	0.812		
Static DCRF	0.914	0.896	0.826		
MES-PFOA-ADCRF	0.931	0.914	0.857		

Statistical Significance Testing (Paired t-Test, α = 0.05)					
Comparisons	Metrics	t-value	p-value	Significance	
Static DCRF vs MES-PFOA-ADCRF	Accuracy	3.84	0.0021	Significant	
Static DCRF vs MES-PFOA-ADCRF	F1-Score	3.57	0.0034	Significant	
Static DCRF vs MES-PFOA-ADCRF	MCC	4.11	0.0016	Significant	
LSTM vs MES-PFOA-ADCRF	Accuracy	4.28	0.0012	Significant	
DNN vs MES-PFOA-ADCRF	Accuracy	5.02	< 0.001	Significant	

### Statistic vs adaptive deep CRF performance comparison

The comparative performance analysis among the static CRF and adaptive DCRF techniques is provided in the following Table 6. The accuracy value attained from the adaptive DCRF approach is 94.81%, which is more extensive than the static DCRF of 90.26%. This highlights that the adaptive model updating process improves the access control decision making efficiency. Moreover, the highest values of the sensitivity and specificity scores ensure the reliability of the proposed adaptive deep CRF model in healthcare applications while making decisions over access control.

**Table 6:** Performance Comparison in terms of Static vs Adaptive Deep CRF.

Performance measures	Static DCRF	Adaptive DCRF	Description
Accuracy (%)	90.26	94.81	Adaptive tuning improves classification boundaries
Specificity (%)	93.33	96.70	Better negative class discrimination
FPR (%)	6.67	3.30	Reduced false alarms due to optimized parameters
FNR (%)	14.06	7.94	Significant reduction in missed diagnoses
FDR (%)	9.84	4.92	Improved decision reliability
F1-Score (%)	88	93.55	Balanced precision–recall improvement
MCC (%)	0.799	0.892	Stronger correlation between predictions and labels
CSI (%)	78.57	87.88	Higher classification success index

### Performance comparison against recent tuned deep learning models

The performance assessment across diverse deep and machine learning approaches for detecting intrusions is given in Table 7. Traditional intrusion detection methods, including CNN, LSTM, SVM and OptCNN-LSTM, are considered for validating the security performance of the secure environment based on the K-fold measure. The accuracy of our MES-PFOA-ADCRF is 92.21% for the K-fold value 1, which surpasses the classical approaches as 6.69% than CNN, 4.84% than LSTM, 2.76% than SVM and 1.46% than OptCNN-LSM. The performance assessment on various K-fold measures ensure that the proposed ADCRF model attained better decision making over access control in a healthcare environment, and hence the security of the sensitive information of the patients is highly protected.

**Table 7:** Performance Assessment across Diverse Machine/Deep Learning Models.

Terms	CNN^48^	LSTM^49^	SVM^50^	OptCNN-LSTM^51^	MES-PFOA-ADCRF
K-fold 1					
Accuracy	86.42	87.95	89.73	90.88	92.207792
Specificity	89.34	90.76	92.58	93.91	94.505495
FPR	10.66	9.24	7.42	6.09	5.4945055
FNR	17.96	16.11	13.84	12.97	11.111111
FDR	14.88	13.45	10.92	9.34	8.1967213
F1-score	83.72	84.96	87.88	88.94	90.322581
MCC	0.7241	0.7419	0.7936	0.8128	0.8383423
CSI	72.13	73.94	77.86	79.44	82.352941
K-fold 2					
Accuracy	88.96	89.88	91.56	92.04	94.155844
Specificity	91.92	92.84	93.77	94.11	95.652174
FPR	8.08	7.16	6.23	5.89	4.3478261
FNR	14.61	13.02	11.94	11.21	8.0645161
FDR	11.94	10.48	9.02	8.41	6.557377
F1-score	86.92	87.88	89.61	90.14	92.682927
MCC	0.7814	0.8037	0.8265	0.8421	0.8782664
CSI	76.88	78.44	80.93	82.11	86.363636
K-fold 3					
Accuracy	86.19	88.74	90.12	91.96	93.506494
Specificity	90.77	92.15	93.01	94.82	95.604396
FPR	9.23	7.85	6.99	5.18	4.3956044
FNR	19.27	15.44	13.79	11.48	9.5238095
FDR	13.62	11.18	9.94	7.42	6.557377
F1-score	82.91	86.47	88.12	90.63	91.935484
MCC	0.7116	0.7743	0.8012	0.8368	0.865346
CSI	71.44	76.92	79.66	82.94	85.074627
K-fold 3					
Accuracy	85.71	86.84	88.96	89.77	90.25974
Specificity	89.62	90.88	92.64	93.12	93.333333
FPR	10.38	9.12	7.36	6.88	6.6666667
FNR	19.84	18.02	15.36	14.88	14.0625
FDR	14.92	13.26	10.88	10.11	9.8360656
F1-score	82.33	83.79	86.92	87.64	88
MCC	0.7062	0.7285	0.7729	0.7914	0.7987636
CSI	70.58	72.41	76.92	77.88	78.571429
K-fold 5					
Accuracy	89.41	91.36	89.02	91.94	94.805195
Specificity	92.11	93.84	92.67	94.91	96.703297
FPR	7.89	6.16	7.33	5.09	3.2967033
FNR	14.96	11.24	15.18	11.62	7.9365079
FDR	11.48	9.22	11.02	7.11	4.9180328
F1-score	86.94	89.88	87.36	90.84	93.548387
MCC	0.7819	0.8264	0.7841	0.8469	0.8923497
CSI	77.36	81.94	77.02	82.96	87.878788

### Comparative assessment of blockchain-based security schemes in healthcare

The security performance of the developed blockchain-based SDN approach and access control mechanism has been validated with the traditional blockchain security methodologies, and the results are provided in Table 8. Classical blockchain platforms like Ethereum (POS) and Ethereum (PoW) attained higher security levels of 90% and 89.2%, respectively. The transaction cost of these models is higher when compared to the token-based access control and lightweight, scalable blockchain mechanisms. The latency of the Ethereum approaches is higher when compared to the conventional IOTA and BlendCAC models used for the validation. When compared to other blockchain-based paradigms, our approach attained effective security performance with low latency and cost, which proves the better data transfer efficiency of the model in healthcare applications.

**Table 8:** Performance Evaluation over Blockchain-based Security Mechanisms.

Measures	Security (%)	Cost per Transaction (USD)	Transactions per Second (TPS)	Transaction Latency (ms)
Ethereum (PoW)	90	4.32	14	65,000
Ethereum (PoS)	89.2	0.48	90	12,000
Hyperledger Fabric (Standalone blockchain model)	88.7	0	2000	820
IOTA (Tangle)	85.6	0	1500	450
BlendCAC	83.9	0.12	250	3400
TBAC (Token-Based Access Control)	82.4	0.21	120	4800
LSB (Lightweight Scalable Blockchain)	84.8	0.08	600	1900
DPoS-based IoT Blockchain	87.3	0.03	1200	9
OACS (Healthcare IoT)	86.1	0.15	20	260
Proposed MES-PFOA-ADCRF (SD-WBAN)	89.6	0.02	850	620

### Latency, throughput and computational overhead analysis

The experimental evaluation of the developed model in terms of diverse measures has been provided in Table 9. The end-to-end latency analysis demonstrates that our integrated blockchain SDN with a machine learning model attains a greater performance in terms of inference delay as 13.1 ms, which is more impressive than the RF, DNN and LSTM approaches. The throughput rate obtained from our MES-PFOA-ADCRF model is 472kbps, which proves the effective reliability and bandwidth efficiency of the developed approach in healthcare applications. The validation with the blockchain measures also proves the better balance of resource efficiency in blockchain-enabled WBAN systems.

**Table 9:** Latency, throughput and Computational overhead analysis.

Latency Analysis					
Components	RF	DNN	LSTM	Static DCRF	Adaptive MES-PFOA-ADCRF
Feature Extraction Delay (ms)	4.2	5.6	6.8	7.1	7.4
Inference Delay (ms)	8.9	12.4	15.7	14.2	13.1
SDN Decision Delay (ms)	5.12	5.28	5.19	5.3	5.3
Blockchain Validation Delay (ms)	404	400	408	410	410
End-to-End Latency (ms)	428.4	433.3	437.8	436.6	435.8

Throughput Impact Analysis					
Metrics	No Security	Static DCRF	MES-PFOA-ADCRF		
Raw WBAN Throughput (kbps)	520	498	492		
Effective Data Throughput (kbps)	520	461	472		
Throughput Degradation (%)	0	11.35	9.23		
Packet Loss Rate (%)	1.8	3.6	2.9		
Network Utilization (%)	91.2	86.4	88.1		

Computational Overhead Analysis					
Controller-Side Overhead					
Models	CPU Utilization (%)	Memory Usage (MB)	Inference Load (ops/s)		
RF	14.2	312	1950		
DNN	21.6	488	1420		
LSTM	26.8	612	1080		
Static DCRF	24.3	545	1260		
MES-PFOA-ADCRF	25.7	568	1310		

Sensor-Node Overhead					
Metrics	Static DCRF	MES-PFOA-ADCRF			
CPU Load (%)	7.4	7.9			
Memory Usage (KB)	214	226			
Energy Consumption (mJ/sample)	1.62	1.68			

Communication Overhead of Blockchain Integration					
Avg. Blockchain Transactions per Minute	Avg. Transaction Size (Bytes)	Communication Cost per Transaction (KB)	WBAN Bandwidth Utilization (%)	Control Message Overhead (%)	
18	342	0.33	4.8	6.1	

Blockchain-Specific Performance Metrics					
Metrics	Ethereum (PoS)	Hyperledger Fabric	Proposed MES-PFOA-ADCRF		
Transaction Confirmation Time (ms)	12,000	820	620		
Transactions per Second (TPS)	90	2000	850		
Blockchain Growth (MB/day)	145	28	34		
Gas / Transaction (Gwei)	42,000	N/A	18,400		
Cost per Transaction (USD)	0.48	0	0.02		

### Scalability validation

**Fig. 11:**
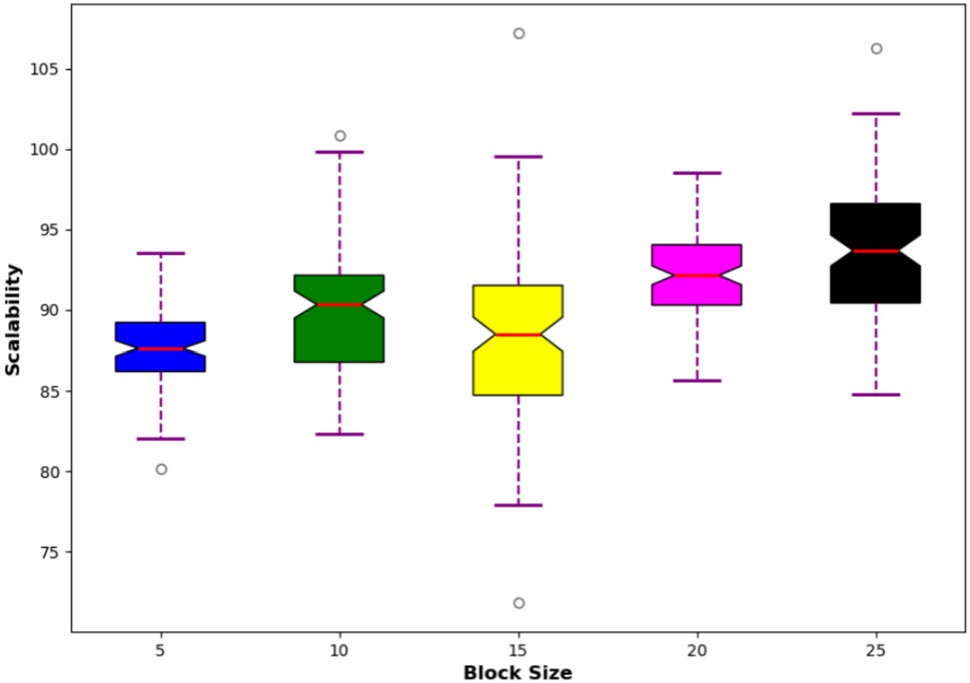
Scalability Validation in a Designed Secure Data Sharing Model.

Scalability analysis executed in the developed secure data sharing model is presented in Fig. 11. In this phase, the scalability of the developed framework is verified by varying the block size. Here, this validation helps to verify how the developed technique effectively handles the communication protocols and enormous data samples at the validation phase. In the validation, the developed MES-PFOA-ADCRF achieved better scalability as 94.5% than the classical techniques. Furthermore, this validation supports ensuring the overall reliability and also supports for precise data distribution.

### State-of-the-art analysis on recent techniques

In this phase, state-of-the-art validations executed in the developed secure data sharing model over the recent techniques is represented in Table 10. Various recently designed techniques considered for the validation are DTAC-TL-QM, SCCE-DS, BFL-hIoT and PPFL-ICP. Performing various analyses in the recently designed techniques helps to verify the overall efficiency of the developed MES-PFOA-O-MA-ABE + ADCRF-based secure data sharing model. In the accuracy validation, the designed MES-PFOA-O-MA-ABE + ADCRF accomplish superior performance as 98.7%, which is higher than the recent techniques. Accomplishing higher accuracy aids in reducing errors and aids in better communication in different environmental conditions. In the encryption time analysis, the designed MES-PFOA-O-MA-ABE + ADCRF are capable of acquiring the data within 210 ms, and also gained a higher throughput as 275 TPS. Improving the throughput in the designed MES-PFOA-O-MA-ABE + ADCRF-based secure data sharing technique offers higher decision-making efficiency and also eliminates the overhead issues.

**Table 10:** State-of-the-art Validation on Recent Techniques in Secure Data Sharing Models.

Recent Techniques	DTAC-TL-QM^29^	SCCE-DS^30^	BFL-hIoT^31^	PPFL-ICP^32^	Proposed MES-PFOA-O-MA-ABE + ADCRF
Accuracy (%)	93.2	94.1	95	95.6	98.7
Precision (%)	92.8	93.7	94.6	95.1	98.3
Recall (%)	92.5	93.3	94.2	94.8	98
F1-Score (%)	92.6	93.5	94.4	94.9	98.1
Encryption Time (ms)	410	380	350	330	210
Throughput (TPS)	165	182	195	205	275

### Discussions

*Real-world Deployment Complexity and Cost of Blockchain*. Though the blockchain are widely used for securing sensitive data samples, they face several shortcomings when implementing, and its details are given as follows. *Deployment complexity*: Generally, the SDWBAN-based controllers are needed to overcome the gaps between the computationally intensive blockchain models and resource-constrained body sensors. A common issue that takes place in blockchain deployment in real-world conditions is poor throughput and higher latency issues. Several delays take place in the network while transmitting the data samples, which affects the overall decision-making in the dynamic scenarios. In some cases, managing the huge amount of data samples is challenging, and it leads to storage issues. *Deployment Cost:* In real-world conditions, deploying the blockchain is expensive according to the requirements of the EHR systems. Moreover, their transaction fee is high for all actions in the public networks. In addition, higher computational power is essential to execute the transaction process. Moreover, the blockchain needs to handle the huge transactions among highly secured applications. In this research, a lightweight blockchain is used, which helps to minimize the transaction cost and computational overhead issues. Using lightweight encryption techniques for secure data sharing makes the framework highly suitable to use in the SDWBAN environment and ensures secure data sharing with manageable operational cost and infrastructure.

## Conclusion

A secure data-sharing system was introduced by employing deep learning techniques along with encryption and optimization techniques. This model employed the system attributes as the input data for performing secure data transmission on the SDWBAN model. Initially, access control was made by employing blockchain technology. Following access control, decision-making was executed by employing the designed ADCRF model to allow only the authenticated user to access the patient’s private information. Furthermore, the decision-making competence of the introduced system was enhanced by tuning the hyperparameters within the DCRF by utilizing the implemented MSE-PFOA technique. In addition, the privacy of the healthcare data was guaranteed by employing an encryption technique. Here, the O-MA-ABE-based encryption model was utilized, where the optimal key was selected by utilizing the developed MSE-PFOA tuning approach. Additionally, the competence of the suggested system was investigated with the standard model and the results revealed that the suggested MES-PFOA-ADCRF decision-making model was 4.28%, 2.09%, 5.03% and 2.09% more effective than RF, DNN, LSTM and DCRF as well as the encryption technique was 25%, 21.05%,6.25%, and 5.66% advance than ECC, RSA, DES, and, MA-ABE. Thus, the suggested secure data-sharing model was more advanced than conventional models in promoting secure data-sharing on the SDWBAN by preventing the risk of unauthorized access. Furthermore, a future enhancement can be executed by considering a few improved security characteristics to perform effective threat detection on the SDWBAN, which assists in boosting the competence of SDWBAN models.

In future works, several extensions need to be considered to support real-time SDWBAN applications that include lightweight deep learning architectures and energy-efficient optimization techniques. The decision-making ability over ADCRF is enhanced with the integration of federated learning and transfer learning techniques, which greatly preserves the healthcare data. Implementation of efficient key revocation schemes during encryption ensures long-term security against evolving attack patterns.

*Applications of Developed Model:* The designed technique is employed for providing secure data sharing in the SDWBAN, and it is also widely applied in different smart healthcare environments. The secure data sharing model is widely used for telemedicine, wearable medical devices, remote patient monitoring and hospitals. As the architecture is designed by considering the SDN, blockchain and attributes-aided encryption procedure, they are widely adapted for other IoT-based data sharing models like environmental monitoring, traffic monitoring, smart cities and supply chain management.

*Ideas from Overall Research:* A secure data sharing model is designed in this research by incorporating blockchain technology with the decision-making procedure. In order to improve the access control, data integrity, and security in the IoT-based healthcare models optimized encryption procedures are used. Moreover, the developed technique uses a secure and scalable procedure for adapting various applications like secure data sharing models, IoT-aided medical systems and smart healthcare monitoring.

## Data Availability

The dataset needed for performing secure healthcare data transfer is attained from the link: "https://www.kaggle.com/datasets/akshaydattatraykhare/diabetes-dataset" with access date 23–01-2026. The name of the dataset is Diabetes Dataset, which is taken from the National Institute of Diabetes and Kidney Diseases. This is a publicly accessible dataset. This dataset is generally used to predict whether the patient has diabetes. This dataset includes attributes such as insulin level, blood pressure, glucose level, age, skin thickness and BMI. From this dataset, 25% of the dataset is used for testing and 75% of the dataset are used for training process. Accessible code for the developed model has been provided in the link "https://github.com/sumanthvmani/Decision-Making-using-ADCRF-with-Encryption/tree/main".

## Abbreviations

ABE: Attribute-Based Encryption
ACL: Access Control List
ADCRF: Adaptive Deep Conditional Random Field
AHISM-B: Advanced Hierarchical Identity-based Security Mechanism by Blockchain
AOA: Addax Optimization Algorithm
API: Application Programming Interface
AVISPA: Automated Validation of Internet Security Protocols and Applications
B-DQCM: Blockchain-Enabled Deep Quantum Computing Model
BMC-SDN: Blockchain-based Multi-Controller with SDN
BS: Body Sensors
BFL-hIoT: Blockchain-enabled Federated Learning for healthcare IoT
BSDNSR: Blockchain-based SDN-aided Secure Routing
BTFAT: Blockchain Technology-Featured Air-Cracking Tool
BUDTP-RPR: Blockchain User Datagram Transport Protocol with Reinforcement Projection Regression
CPA: Chosen Plaintext Attack
CRF: Conditional Random Field
CSI: Channel State Information
DES: Data Encryption Standard
DCRF: Deep Conditional Random Field
DLT: Distributed Ledger Technology
DNN: Deep Neural Network
DS-Contract: Data-Sharing Contract
DTAC: Dynamic Trust-Aware Controller
ECG: Electrocardiogram
ECC: Elliptic Curve Cryptography
EHR: Electronic Health Records
FDI: False Data Injection
FDR: False Discovery Rate
FNR: False Negative Rate
FPR: False Positive Rate
FSA: Flamingo Search Algorithm
6G-IoNMT: 6G with Internet of Nano Medical Things
IoT: Internet of Things
IPFS: Inter-Planetary File System
KPA: Key-Related Attack
LSTM: Long Short-Term Memory
MA-ABE: Multi-Authority Attribute-Based Encryption
MES-PFOA: Modified Escape Search-based Piranha Foraging Optimization Algorithm
MCC: Matthews Correlation Coefficient
MITM: Man-In-The-Middle
MRA: Mud Ring Algorithm
NBI: Northbound Interface
O-MA-ABE: Optimal Key-based Multi-Authority Attribute-Based Encryption
P2P: Peer-To-Peer
PBFT: Practical Byzantine Fault Tolerance
PPFL-ICP: Privacy-Preserving Federated Learning with Intelligent Control Policy
RCM: Route Correctness Mechanism
RF: Random Forest
RSA: Rivest–Shamir–Adleman
SCCE-DS: Secure and Controllable Cloud–Edge Collaborative Data Sharing
SDN: Software-Defined Networking
SDESW: SDN-aided Switches
SDWBAN: Software Defined Wireless Body Area Network
SBI: Southbound Interface
TL-QM: Threshold-based Logic with a Quarantine Mechanism
WBAN: Wireless Body Area Networks
WiFi: Wireless Fidelity
